# Structure and Reactivity of Active Oxygen Species on Silver Surfaces for Ethylene Epoxidation

**DOI:** 10.1021/acscatal.4c01566

**Published:** 2024-06-21

**Authors:** Man Guo, Nanchen Dongfang, Marcella Iannuzzi, Jeroen Anton van Bokhoven, Luca Artiglia

**Affiliations:** † Laboratory for Catalysis and Sustainable Chemistry, Paul Scherrer Institute, 5232 Villigen, Switzerland; ‡ Department of Chemistry and Applied Biosciences, Institute for Chemical and Bioengineering, ETH Zurich, 8093 Zurich, Switzerland; § Department of Chemistry, University of Zurich, 8057 Zurich, Switzerland

**Keywords:** ethylene epoxidation, ambient pressure X-ray photoelectron spectroscopy, oxygen activation, density functional theory, binding energy simulations, time-resolved photoelectron spectroscopy

## Abstract

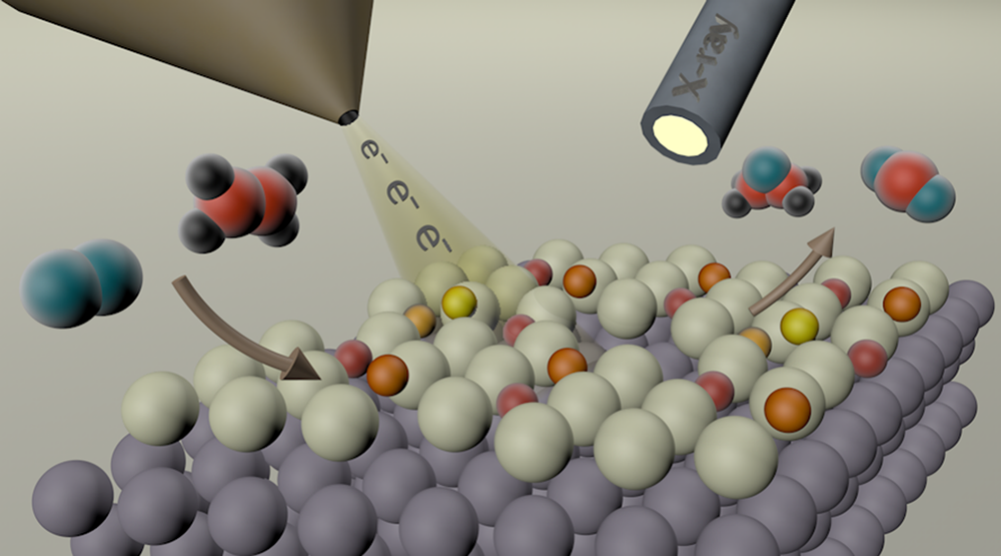

The epoxidation of ethylene stands as one of the most important industrial catalytic reactions, and silver-based catalysts show superior activity and selectivity. Oxygen is activated on the surface of silver during the reaction and exerts a substantial impact on product selectivity. Notably, the oxygen species residing in the topmost atomic layers profoundly influence the reactivity of a catalyst. However, their characterization under in situ reaction conditions remains a huge challenge, and specific structures have not been identified yet. In this study, we employ in situ X-ray photoelectron spectroscopy and density functional theory calculations to determine the oxygen species formed at the topmost atomic layers of a silver foil and to assign them a structure. Three different groups of oxygen species activated on silver are identified: (i) surface lattice oxygen and two oxygen species originating from associatively adsorbed dioxygen and (ii) top and (iii) subsurface oxygen. Transient in situ photoelectron spectroscopy experiments are carried out to reveal the dynamic evolution and thus reactivity of the different oxygen species under ethylene epoxidation reaction environments. The top oxygen atom from the adsorbed associated dioxygen is the most active. Meanwhile, a frequency-selective data analysis method, developed to process time-resolved data, provides insights into the evolving trends of peak intensities for different oxygen species. The versatility of this method suggests its potential application in future time-resolved characterization studies.

## Introduction

Ethylene oxide (EO) holds a pivotal role in the chemical industry as a fundamental building block. Its production through ethylene epoxidation (EPO) is a key process in the chemical sector, with silver as the most-frequently used material for this reaction.^1^ Silver-based catalysts currently represent the sole commercially viable EPO catalyst, owing to their high selectivity toward EO. In the actual EPO environment, the adsorption of oxygen molecules onto the silver surface, their activation, and the subsequent oxidation of ethylene, drive the entire catalytic process. The nature of the active oxygen species that form on the surface of silver is a critical determinant of EO selectivity.^2−4^ Industrially, a wide range of promoters is used to favor the selectivity toward EO.^1^ They are added to the catalyst during the synthesis, such as cesium and rhenium,^5^ and they are added to the reactant feed (vinyl chloride).^6^ The effect of promoters is not the topic of this work; however, a clear identification of the oxygen activation paths is of paramount importance to determine the effect of promoters. In this respect, oxygen species residing on the topmost atomic layers play an important role,^7^ and investigating their type and nature under reaction conditions is crucial for comprehending what triggers the selectivity. Nevertheless, such a characterization is challenging because it requires in situ/operando measurements performed with a surface sensitive spectroscopy method.

Over the past half-century, X-ray photoelectron spectroscopy (XPS) has frequently been used to investigate oxygen species on silver catalysts, as outlined in Table S1. Most studies focused on model surfaces, such as single crystals, under ex-situ, high vacuum conditions using various pretreatment protocols. Two different forms of chemisorbed atomic oxygen were detected on Ag (111) after pretreatment with gaseous oxygen in the 10^–2^–1.0 mbar pressure range.^8^ Atomic oxygen species resulting from p(4 × 4) and c(4 × 8) surface oxide reconstructions were identified on Ag(111) pretreated with NO_2_ and atomic oxygen produced via a thermal gas cracker.^9^ Additionally, new O 1s photoemission peaks assigned to molecular oxygen were detected upon exposure of a silver foil to water vapor at 800 °C and to oxygen ions, respectively.^10^ Currently, thanks to the development of electrostatic prelenses and differential pumping stages, routine in situ photoemission measurements in the mbar pressure range have become feasible in both synchrotron facilities and laboratory setups.^11−14^ Initial attempts have also been made to extend measurements to the bar pressure range.^15^ A few synchrotron radiation-based ambient pressure (AP)-XPS studies have been carried out on silver-based materials under in situ EPO conditions. The analysis of O 1s spectra of a silver powder exposed to a mixture of ethylene and oxygen at 0.3 Torr revealed two main types of oxygen species, identified as electrophilic oxygen and nucleophilic oxygen. Electrophilic oxygen was correlated to the production of ethylene oxide (EO).^16^ Electrophilic oxygen has subsequently been assigned to sulfate adsorbed on silver, whose formation is due to sulfur impurities.^4^ Recent studies performed codosing C_2_H_4_ and O_2_ on a silver nanopowder and on a Ag/Al_2_O_3_ catalyst identified several active oxygen species at the surface.^2,3^ In situ Raman results showed the following features: (i) surface O* species and Ag–O_bulk_ species (lattice oxygen) exhibiting Ag–O vibrations in the 300–500 cm^–1^, (ii) surface dioxygen species (O_2_*) adsorbed on silver exhibiting O–O vibrations in the 600–800 cm^–1^ range (Ag_4_–O–O), and (iii) surface molecular species (O_2_*) adsorbed on silver exhibiting O=O vibrations in the 1000–1200 cm^–1^ range (Ag_2_*O=O). Finally, dioxygen species (Ag_*x*_–O_2_) were proposed to selectively oxidize ethylene based on density functional theory (DFT) calculations combined with in situ Raman. A novel approach proposed by Chen et al., making use of advanced machine-learning grand canonical global structure simulations and in situ infrared experiments, identified square-pyramidal subsurface oxygen on Ag(100) as the selective phase for ethylene selective oxidation.^17^ In summary, a systematic investigation into the clear structures of oxygen species within the topmost atomic layers of silver under in situ conditions is still lacking. Such research is essential to understand the roles of different types of oxygen species in EPO and eventually the effect of promoters/dopants on them to enhance the selectivity toward EO.

This study reports a thorough in situ AP-XPS investigation of ethylene oxidation on silver foil. The photoemission signals of O 1s and Ag 3d were acquired in high resolution under steady-state conditions at various ethylene and oxygen partial pressures from room temperature to 400 °C. DFT calculations of the oxygen core electron binding energy for different species combined with depth-profile analyses performed at different kinetic energies provide a clear classification of the stable structures present on the surface of silver at 1 mbar. We identify three distinct types of silver-related oxygen, which could be the active species in the EPO reaction: surface lattice oxygen and top and subsurface oxygen from the associated adsorbed dioxygen.

Finally, we perform transient response experiments at different temperatures and develop a frequency-selective data analysis method (FSDA) to process the time-resolved AP-XPS data and extract the signals that change over time. This allows us to distinguish the role of the different oxygen species in the reaction process. The top oxygen from adsorbed associated dioxygen are the most active. Therefore, the properties of these species have a significant impact on the catalytic activity. The FSDA method can generally be applied to process time-resolved photoemission data.

## Methods

AP-XPS measurements were carried out at the X07DB in situ spectroscopy beamline at the Swiss Light Source (SLS) synchrotron. A silver foil (Alfa Aesar, 99.99%) was cut into 10 × 10 mm squares and cleaned by solvents (acetone, isopropanol and water), followed by plasma cleaning cycles under oxidizing (oxygen–argon mixture) and reducing (hydrogen–helium mixture) atmosphere in order to remove almost quantitatively carbon and oxygen impurities from the surface. Clean foils were fixed to a manipulator and introduced into the solid–gas interface endstation, which allows for precise dosing of gas/gas mixtures under flow conditions.^12,18^ Before starting the in situ experiments, the sample was heated in vacuum to 400 °C to remove all the adsorbates (the cleanliness was checked by means of XPS). Gas mixtures were dosed by means of mass flow controllers. Gases were pumped away with a tunable diaphragm valve placed downstream the cell and connected to a root pump. This allows for the dosing of gas flows while precisely controlling their partial pressures and thus ratio during the experiments. The pressure was monitored by means of Baratron measurement heads. The samples were heated using a tunable infrared laser hitting the back of the sample holder, and the temperature was monitored by means of a Pt100 sensor. The sample was investigated by acquiring all the photoemission peaks in sequence, while being exposed to a specific gas/gas mixture at different temperatures. Linearly polarized light was used throughout the experiments. Ag 3d peaks were acquired with photon energies (*h*ν) of 575 and 855 eV, corresponding to kinetic energies of ∼200 and 480 eV. Such values were chosen to explore the sample compositions at increasing depth. The inelastic mean free paths (λ) of Ag 3d photoelectrons at the two explored kinetic energies are 5.29 and 8.79 Å.^19^ The corresponding mean escape depths (MED – defined as λ × cos(θ), where θ = 30°, due to the geometry of the sample surface with respect to the analyzer) are 4.58 and 7.61 Å. O 1s peaks were acquired with *h*ν = 735 and 1015 eV, corresponding to the same kinetic energies used for Ag 3d, thus providing information about the same MEDs. The BE scale was aligned using the 3d peak of metallic silver, centered at 368.2 eV, as a reference.^20,21^ After subtraction of a Shirley background, the photoemission peaks were fitted by Voigt-shaped functions, and the fitting parameters are reported in Tables 1, S2–S4.

**Table 1:** Fitting Parameters of O 1s Peaks (K.E. = 200 eV)

	BE (eV)	fwhm (eV)	%L-G
O_C1_	533.7	1.3	25%
O_C2_	532.8	1.4	25%
O_C3_	532.1	1.4	25%
O_S_	531.5	1.5	25%
O_T_	530.7	1.5	25%
O_L_	529.3	1.4	25%

All DFT calculations were performed using the Quickstep module of the CP2K (Development version) program package.^22^ For geometrical optimization and electronic structure calculations, the Kohn–Sham DFT within the hybrid Gaussian and plane waves framework (GPW) was applied, with Goedecker-Teter-Hutter (GTH) pseudopotentials.^23^ The molecular orbitals of the valence electrons are expanded in molecularly optimized (MOLOPT) Gaussian basis sets.^24^ DZVP-MOLOPT-GTH primary basis sets were used for Ag and O. For the auxiliary plane waves basis set, a cutoff of 600 Ry was applied. All calculations were done within periodic boundary conditions, at Gamma point only, and were spin-polarized. Since we used an asymmetric slab model, where the oxidized-reconstructed surface is only on one side, the surface dipole correction was always applied along the *z*-axis.^25^ Structural and electronic properties were computed at the GGA level of theory, using the Perdew–Burke–Ernzerhof (PBE) functional,^23^ augmented by the Grimme-D3 scheme to correct for the missing dispersion contributions.^26^ The geometry optimization applies the Broyden-Fletcher-Goldfarb-Shanno (BFGS) scheme,^27^ with a force threshold of 10^–3^ Hartree/Bohr. To accurately replicate various oxygen species on an Ag foil, our approach involved considering both the clean Ag (111) surface and oxidized-reconstructive surface structures. As reported in the introduction section, a recent study demonstrates that the most selective oxygen phase toward EPO forms on Ag(100).^17^ However, among the low index facets, (111) is the most thermodynamically stable, as shown by surface energy calculations;^28^ thus, it represents well the behavior of silver nanoparticles on the actual catalyst. We focused our work on Ag(111) because the purpose was to simulate the silver surface model which was constructed based on STM studies conducted under conditions similar to those employed in our work, ensuring a realistic representation.^9,29,30^ In particular, Andryushechkin et al. pretreated Ag (111) with O_2_ under 1 Torr at different temperatures and found that the single crystal surface remained unreconstructed at RT and tended to form p(4 × 4) reconstructions at 200 °C.^29^ Martin et al. pretreated Ag (111) with atomic oxygen and demonstrated that various reconstructions, primarily p(4 × 4) and c(4 × 8), were copresent on the surface.^9^ Thus, the thermodynamically stable (111) termination of metallic silver and the two major reconstructed surface oxides, p(4 × 4) and c(4 × 8), are considered in the current work, and the substrates are shown in Figure S11. These structures were optimized at the DFT level, and the electronic structure, specifically the core BE of different molecular and atomic oxygen species, was calculated. The constructed model involved a 5-layer Ag(111), with the top layer modified by introducing O atoms to create p(4 × 4) and c(4 × 8) oxidized reconstructions. Based on these reconstructions, additional atomic oxygen was introduced to interact with existing oxygen atoms on the surface, forming various dioxygen species on the oxidized Ag(111) surface. The lateral dimensions of the simulation cell are 23.19 × 23.19 Å^2^. The sufficient vacuum space (20 Å) both above and below the slab prevents spurious physical interactions among the periodic images.

The calculation of the O K-edge, used to reproduce the binding energies of 1s electrons, was achieved by applying the Gaussian augmented plane wave method (GAPW). For the O elements, all electrons were explicitly considered (no pseudopotentials) and 6-311G** all-electron basis sets were employed to expand the molecular orbitals. The calculations of the energies of the core states were based on the Slater transition potential method with half-core hole approximation, where initial and final state effects were accounted for by electronic energy eigenvalue calculations after removing half an electron from the core state.^31,32^

## Results and Discussion

### Oxygen Activation on the Surface of Silver Foil under EPO Conditions

**Figure 1:**
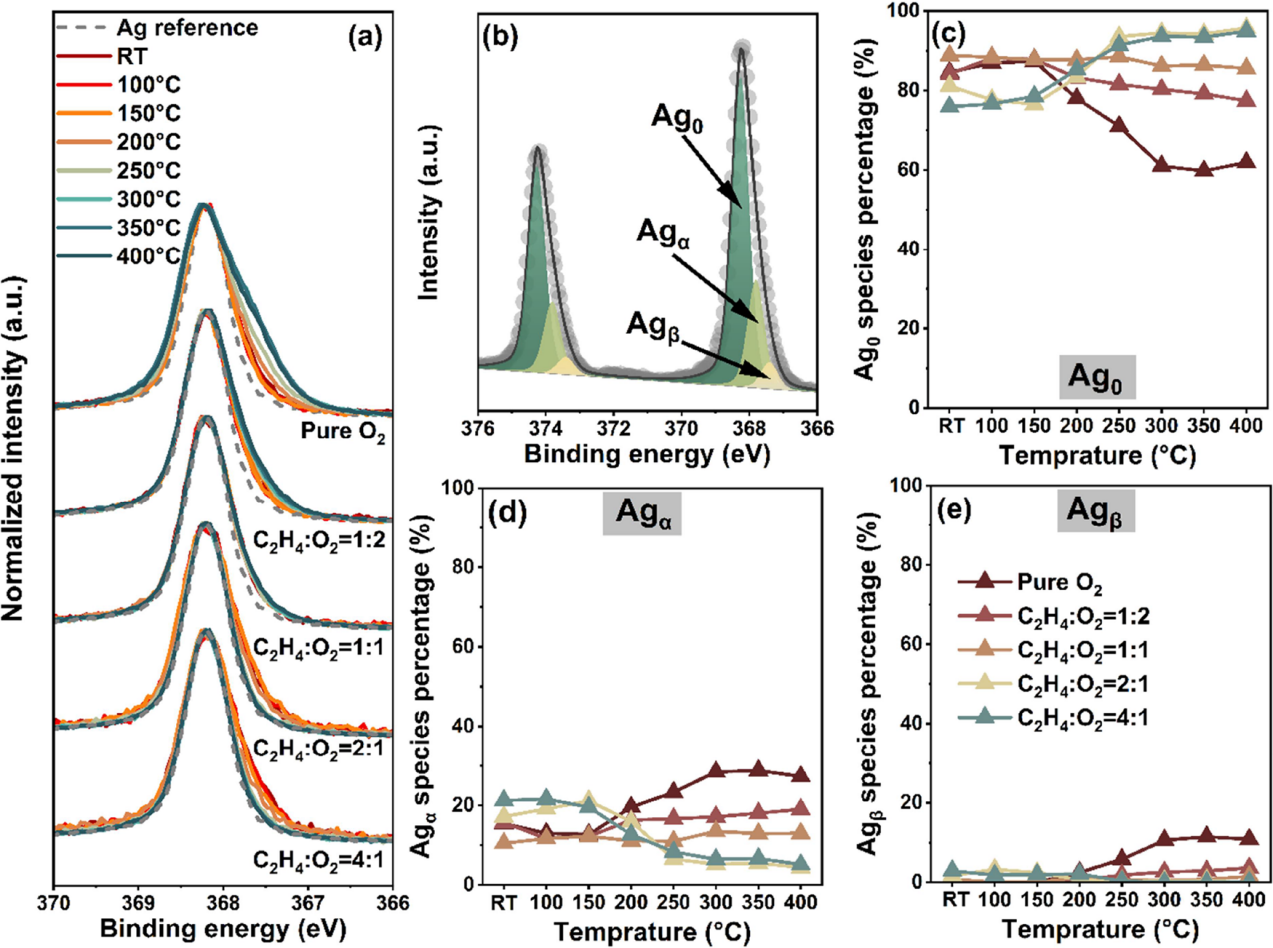
Experimental Ag 3d spectra recorded under different gas conditions and temperatures and their deconvolution. (a) Ag 3d_5/2_ spectra acquired from room temperature to 400 °C and under different ethylene: oxygen ratios (K.E. = 200 eV). The dashed line spectrum was measured under high vacuum at 400 °C as a reference. (b) Example of Ag 3d deconvolution. (c–e) Fractions of Ag_0_ (c), Ag_α_ (d), and Ag_β_ (e) in different reaction environments as a function of temperature.

Figure 1a illustrates the Ag 3d spectra normalized to the maximum measured at 1 mbar total pressure under different C_2_H_4_/O_2_ ratios while heating the sample from room temperature to 400 °C. Prior to these measurements, reference Ag 3d high resolution spectra were obtained under high vacuum (HV) conditions at 400 °C after introducing a silver foil to the chamber, as depicted in Figure S1. At this stage, the silver foil surface is fully reduced (hereafter named Ag_0_) and displays a single doublet (Ag 3d_5/2_ centered at 368.2 eV). The Ag 3d spectrum collected at 200 eV kinetic energy (KE - Figure S1a) exhibits a narrower full width at half-maximum (fwhm) value (0.6 eV) than that of the spectrum collected at 480 eV (1.0 eV). This is mainly due to the change in energy resolution (resolving power of the beamline monochromator) at the two photon energies used. The fitting parameters extrapolated from reference spectra (Table S2) were used for the deconvolution of Ag 3d spectra shown in Figure 1. As depicted in Figure 1a, a peak shoulder at lower BE is evident in the spectra recorded under all conditions compared to the spectrum recorded in vacuum (gray dashed line). Two components, centered at 367.8 eV (Ag_α_) and 367.4 eV (Ag_β_), are identified at both 200 and 480 eV kinetic energy (Figures 1b, S2 and S3, Table S3). Reference spectra of Ag_2_O and AgO reported in the literature display BEs of 367.7 and 367.3 eV, respectively; thus, the new peaks are tentatively assigned to AgO_*x*_.^33,34^ The low intensity peak centered around 372.0 eV is assigned to final state shakeup satellites, which have been discussed in the literature.^35,36^ The samples used in this study are silver foils and thus not comparable to silver oxide reference samples. As shown in Figures 1c–e and S4, even at 400 °C in pure oxygen, the surface is predominantly composed of Ag_0_. In oxygen excess conditions, both Ag_α_ and Ag_β_ display an increasing trend starting above 150 °C.

**Figure 2:**
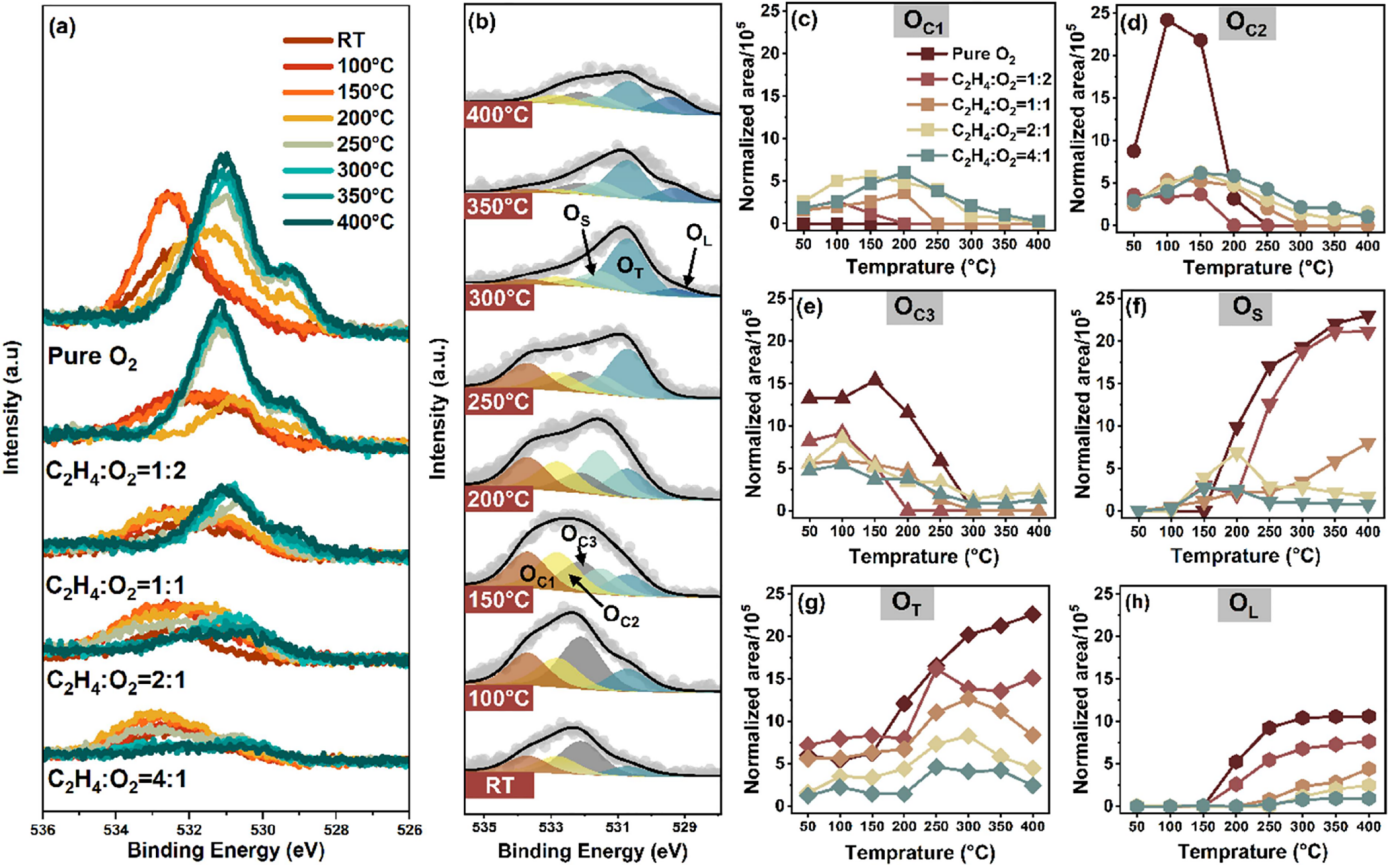
Experimental O 1s spectra recorded under different gas conditions and temperatures and their deconvolution. (a) O 1s spectra acquired in different reaction environments with increasing temperature from room temperature to 400 °C. (KE = 200 eV) (b) O 1s peaks evolution from room temperature to 400 °C (C_2_H_4_:O_2_ = 2:1, KE = 200 eV). (c–h) Fractions of O_C1_ (c), O_C2_ (d), O_C3_ (e), O_S_ (f), O_T_ (g), and O_L_ (h) in different reaction environment as a function of temperature.

**Figure 3:**
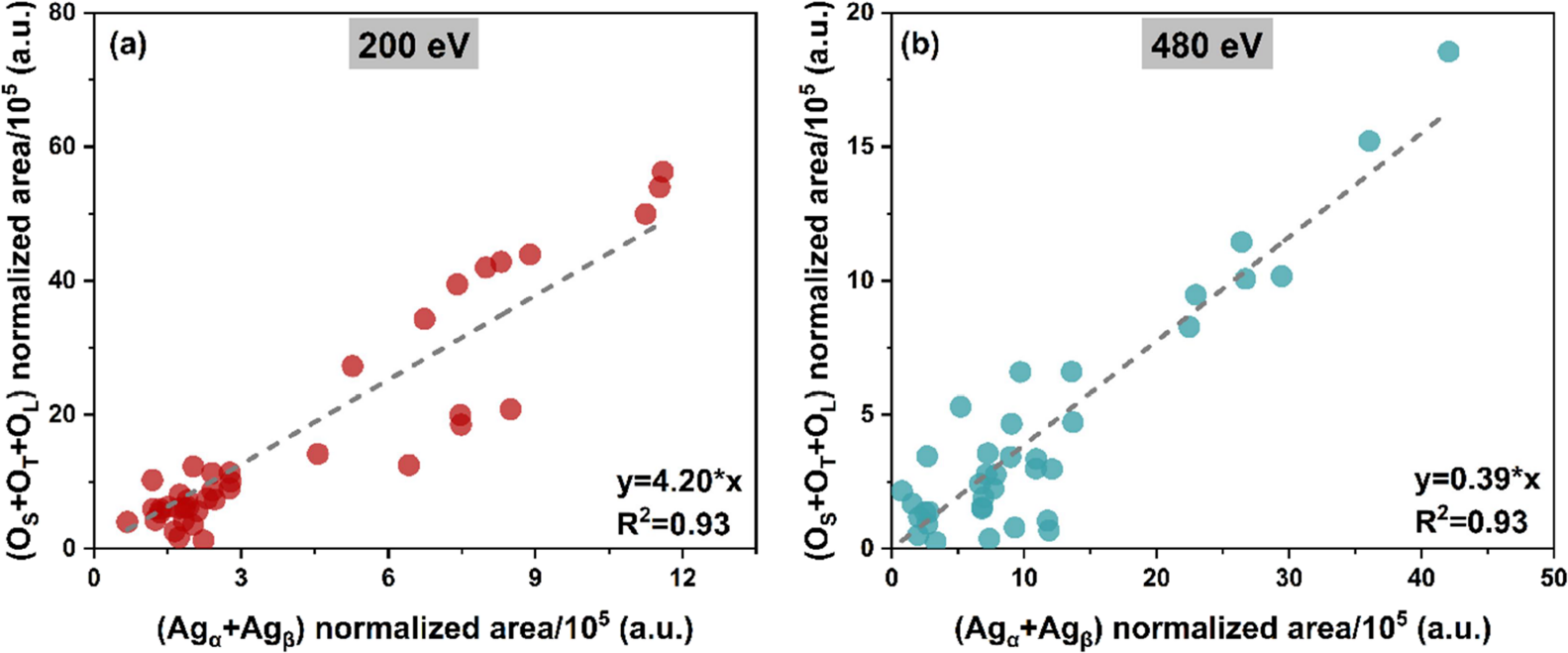
Correlation of the normalized peak areas of O_S_ + O_T_ + O_L_ and Ag_α_ + Ag_β_ at 200 eV (a) and 480 eV (b).

Figure 2a displays the O 1s spectra (KE = 200 eV) collected in situ while exposing the sample to the same reaction conditions as those used for recording Ag 3d spectra. The intensities are normalized by the photon flux, cross section of the O 1s core level, and MED, to compare the content and behavior of oxygen species between the two investigated KEs of 200 and 480 eV. The total intensity of the O 1s spectra gradually decreases with increasing ethylene partial pressure. Six distinct O 1s peak components ranging from 529.3 to 533.7 eV are identified, as shown in Figures 2b and S5–S6, and the corresponding fitting parameters at 200 and 480 eV are listed in Tables 1 and S4, respectively. The oxygen species can be categorized into two main groups based on the trend of the normalized peak area with temperature in different gas conditions (Figures 2c–h and S7). The three oxygen species represented by peaks with BE higher than 532.1 eV are present on the silver surface at room temperature and decrease with temperature, to almost vanish above 200 °C. The three oxygen species represented by peaks with BE lower than 531.5 eV gradually form on the surface as the temperature increases. Based on assignments from previous literature, O 1s having BE values above 532.1 eV are mainly attributed to carbon-containing intermediates.^37−42^ Therefore, we denote them as O_C1_, O_C2_, and O_C3_, respectively. Because the scope of this work is to discuss oxygen reaction intermediates formed on silver during the oxidation of ethylene, we will not discuss in detail these species. O 1s peaks at lower binding energies are denoted as O_S_, O_T_, and O_L_; a clear explanation of the assignments will be provided in the following. As shown in Figure 3, the good correlation between the sum of O_S_, O_T_, and O_L_ and the sum of Ag_α_ and Ag_β_ after normalization to the photon flux, cross section, and MED, confirms that these three oxygen species arise from silver-related oxygen. A targeted analysis was conducted to obtain more information about the depth distribution of O_S_, O_T_, and O_L_. O 1s spectra were collected at kinetic energies of 200 and 480 eV under each experimental condition. As reported in the Methods section, the probing depth of XPS can be tuned by varying the photoelectron kinetic energy (see Figure S8a). The correlation between the normalized peak areas of each O 1s component at 200 eV (*x*-axis) and the same at 480 eV (*y*-axis) is shown in Figure S8b–d. The slopes derived from linear fittings provide information about the depth distribution of different oxygen species. O_L_, centered at 529.5 eV, has the highest slope, indicating that it is distributed more subsurface than the other two species. O_T_ (at 530.7 eV) has the lowest slope; it represents oxygen in the topmost layer. The slope of O_S_ (at 531.3 eV) falls between that of O_L_ and O_T_, suggesting that it is distributed at an intermediate depth.

DFT calculations were further employed to characterize the stable oxygen adsorbates on the surface of silver. Three substrates were simulated, notably Ag(111), and oxidized-p(4 × 4) and -c(4 × 8) reconstructions, considering the adsorption of molecular oxygen and atomic oxygen. The adsorption energy values per oxygen atom, calculated as , where *E*_total_ is the energy of the fully optimized surface with added O atoms, *E*_slab_ is the reference reconstructed surface or the reference Ag(111) surface, depending on the system, and *E*_O2,gas_ is the energy of the optimized O_2_ molecule in the gas phase, are reported in Table S6. They range from −0.33 to −0.58 eV, indicating favorable adsorption. The calculated O 1s electron BEs are obtained for all stable oxygen species and are used to refine the structures associated with the experimental XPS data.

**Figure 4:**
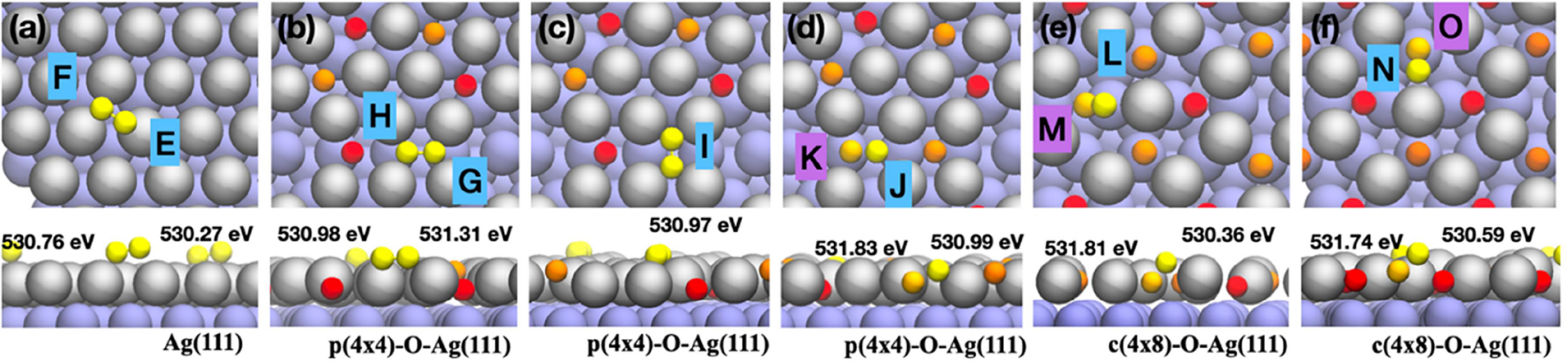
Optimized DFT models on the partially oxidized Ag (111) surface. Optimized structure and calculated BE of oxygen species on unreconstructed Ag (111) facet (a), p(4 × 4) structure (b, c) and c(4 × 8) structure (d–e). The gray and purple spheres are silver atoms on the surface and subsurface, correspondingly. The red, orange, and yellow spheres correspond to O_L_, O_S_, and O_T_.

The most stable adsorption of molecular oxygen on Ag(111) occurs at the FCC site with an energy of −0.35 eV, whereas that of the HCP site is −0.33 eV. The molecule axis is slightly tilted with respect to the surface, as shown in Figures 4a and S9 (displaying also the adsorption of molecular oxygen on HCP sites of Ag(111)) and the O–O bond elongates from 1.21 to 1.40 Å, caused by charge-transfer from the metal to the antibonding orbitals of oxygen, weakening the bond. The oxygen atom that is positioned the closest to the silver surface coordinates with two silver atoms on the bridge site of Ag(111). The other oxygen atom is coordinated to just one silver atom, at a slightly shorter distance. The calculated O 1s BEs are 530.76 and 530.27 eV, respectively, suggesting a slightly electrophilic character. Atomic oxygen preferentially adsorbs into the FCC sites (−0.50 eV) compared to HCP sites (−0.41 eV). The calculated O 1s BE of atomic oxygen is approximately 528.5 eV, comparable to reports from other studies (see Figure S10),^43^ confirming a strong nucleophilic character. However, it is noteworthy that experimental signals below 529.0 eV are absent, suggesting that atomic oxygen adsorbed on an unreconstructed Ag(111) surface is unlikely under the experimental conditions used in this work.

On the p(4 × 4) oxidized reconstructed surface, two types of atomic oxygen are present, as shown in Figures 4b–d and S11a,c. These two species are oxide-like oxygens, whose regular arrangement at the edges of the triangular Ag(111) islands determines the characteristic reconstruction. They are located in layer at the edges of the triangular Ag(111) islands but at slightly different heights above the subsurface silver layer. Hence, the chemical environment is slightly different and the O 1s BEs are calculated to be 529.78 eV for the deeper O (red in the figure) and 529.48 eV for the other one. Also, the c(4 × 8) reconstruction is characterized by two distinct oxygen species, as shown in Figures 4e,f and S11b,d. One site is atop of a subsurface silver atom (orange in the Figure), and the other is at a subsurface layer’s bridge site (red in the Figure). Also, in this case, the different chemical environment corresponds to different computed O 1s BEs, 529.81 and 529.56 eV, respectively. For all these oxide-like oxygen species, involved in the formation of oxygen surface reconstructions, we obtained O 1s BE values ranging from 529.48 to 529.81 eV, which are consistent with the experimentally measured O_L_ spectral component centered at 529.5 eV. The lower O 1s BE indicates that there is significant hybridization between Ag 4d and O 2p orbitals, which is also revealed by projected density of states (PDOS - Figure S12c,d), where the O-p states form an occupied band between −3.0 and −0.5 eV below the Fermi energy. Similar results are also reported by Jones et al.,^44^ where the strongly ionic Ag–O bonding is associated with the nucleophilic character of the oxide-oxygen.

By adding oxygen atoms in the proximity of one oxide-oxygen on reconstructed surfaces, dioxygen species are formed during DFT optimization, where the oxide-oxygen may be partially or fully pulled out from the original in-layer site, as shown in Figure 4b–f (colored in yellow). On p(4 × 4) reconstruction, the upper oxide-oxygen (orange) is extracted from the silver layer and forms a dioxygen species which lays parallel to the surface, either along the edge Ag(111) triangular island (Figure 4b) or across it (Figure 4c). The resulting O 1s BE ranges between 530.97 and 531.31 eV, where the variations are determined by the details of the specific chemical environment. In all cases, the BE values indicate a pronounced electrophilic character. This can be associated with a different amount of hybridization with the silver d-band, with respect to oxide-oxygen. This is also revealed by inspecting the PDOS reported in Figure S13, where a strong peak of localized O p states is present just below the Fermi energy, as a sign of enhanced reactivity. When one oxygen is added in proximity of the lower oxide-oxygen, the resulting dioxygen structure optimizes in a tilted configuration, as shown in Figure 4d. The oxide-oxygen is pulled upward but still maintains an inlayer position; the added oxygen is attracted into a hollow site between two silver atoms. The resulting O–O distance is 1.48 Å, and the BEs are 531.83 and 530.99 eV. The occupied p states of oxygen are pushed at higher energies, forming a peak below the Fermi energy, indicating a reduced hybridization with the d-band of silver. On the c(4 × 8) reconstructed surface, we find in both cases tilted dioxygen configurations, shown in Figure 4e,f. Like on the p(4 × 4), the O 1s BEs corresponding to exposed oxygen species have lower values (530.36 and 530.59 eV) than the deeper ones (BE of 531.81 and 531.74 eV). From the PDOS (Figure S14), the tilting pattern is attributed to the orientation of p states along *x*, *y,* or *z* axis, depending on the bonding environment. It also confirms the enhanced electrophilic character, with respect to oxide-oxygen, where occupied and localized p states are pushed just below the Fermi energy and some empty p states appear just above.

To summarize the findings from DFT calculations, three distinct types of oxygen species are identified on the oxidized silver surface that can be related to the experimentally revealed species. The categorization of oxygen varieties based on the BEs of their O 1s peaks allows for valuable deductions regarding their properties and bonding conditions. Tables S5 and S6 report a comparison of experimental and calculated BEs and a summary of all the calculated adsorption energies and BE values, respectively. Figure S15 displays the reference models of atomic oxygen adsorbed on HCP sites of Ag(111), AgO_2_, and Ag_4_O_4_ reported in Table S6. Among the discussed stable oxygen species, the one displaying the lowest O 1s BE falls within the range of 529.48 to 529.81 eV and corresponds to the oxygen atoms forming two types of oxide reconstructions on the Ag(111) surface, referred to as O_L_ (lattice). This species has a nucleophilic character and can be related to “nucleophilic oxygen” previously reported in the literature.^16^ The O_L_ species correspond to surface O* species previously detected by Raman on a silver powder and on a Ag/Al_2_O_3_ catalyst and simulated by DFT.^2,3^ The other two species detected by means of XPS, which correspond to O 1s BE values higher than 530 eV, can be assigned to dioxygen species formed at the silver oxide surface. Such species display an electrophilic behavior and their properties correlate well with “electrophilic oxygen” identified by XPS and with surface adsorbed dioxygen identified by Raman and DFT.^2,3,16,45^ According to the simulated structures, two distinct signals occur for tilted adsorption configurations: when one oxygen atom is a part of the oxidized silver surface, the BE of O 1s ranges between 530.36 and 530.99 eV, and when oxygen is exposed at the solid–gas interface, the BE varies between 530.76 and 531.83 eV. These species are then identified as O_S_ (subsurface, BE = 531.5 eV) and O_T_ (top, BE = 530.7 eV) detected by XPS. It is worth mentioning that, according to the simulation, the O_T_ signal can also be assigned to molecular oxygen adsorbed on pristine Ag(111). While the exact replication of these models in real experiments may vary, a wide pool of oxygen species adsorbed on the substrates are considered in DFT simulation. The BE trend simulated by DFT (BE_O_L__ < BE_O_T__ < BE_O_S__) correlates well to the depth-profile analysis of the three oxygen components detected by XPS. Finally, nucleophilic and electrophilic oxygen, whose origin has been debated for a long time, is assigned to specific oxygen structures formed on the surface of silver. The charges on the simulated oxygen species, obtained by incorporating data from the Mulliken analysis (Mulliken charge population), are shown in Table S6. According to this analysis, atomic oxygen species embedded in the surface (surface reconstructions) carry a negative charge of about −0.5 e. When molecular species are formed, the charge is reduced to about −0.2 e. This confirms the stronger hybridization with the Ag-d band of the lattice oxygen and further proves the electrophilicity of molecular oxygen species. Interestingly, XPS experiments show that O_T_ is present at room temperature and increases with temperature, whereas O_S_ is detected only above 150 °C, together with O_L_. This demonstrates that metallic silver adsorbs dioxygen at room temperature. The formation of surface oxides starts above 150 °C and further favors the adsorption and activation of dioxygen, as demonstrated by the simultaneous increase of O_T_, O_S,_ and O_L_. Interestingly, DFT results are in fair agreement with XPS plots in Figure 2f–h. Indeed, the calculated BE values for dioxygen adsorbed on metallic Ag(111) are below 531 eV and the BE of O_S_ shifts to values >531 eV only upon adsorption on surface oxides. Another relevant finding of this work is the correlation between Ag 3d (Ag_α_ and Ag_β_) and O 1s (O_L_, O_T,_ and O_S_) in Figure 3. Actual catalysts are made of silver nanoparticles supported on an oxide (e.g., alumina); thus, a main contribution from the support (lattice oxygen) is present in the O 1s. Our results demonstrate that it is possible to follow “indirectly” the activation and evolution of oxygen during the oxidation of ethylene investigating the oxidic components in the signal of Ag 3d acquired in high resolution.

### Frequency-Selective Analysis of Transient Photoemission Spectra

O_S_, O_T,_ and O_L_ were detected by means of steady-state in situ XPS experiments, and their structure was identified by crosschecking with theoretical calculations. Steady-state experiments are useful to get an overview about the local electronic state of species and their depth distribution. However, during a steady-state acquisition, the collected O 1s signal encompasses both active species, which participate in the reaction, and spectator species, which reside on the surface without actively participating in the reaction. To identify differences in reactivity of the adsorbed species, a series of time-resolved transient experiments were conducted. Such transient experiments were executed in the same setup as the steady-state experiment, with the reaction environment being perturbed by periodic pulses.^46−48^ During the experiment, ethylene and oxygen with a ratio of 2/1 were first dosed to the cell for 8 min; then, oxygen was replaced by the same partial pressure of argon. In the meantime, the O 1s signal was acquired in fast scan mode with a time resolution of 5.0 s and the intensities of carbon dioxide and EO were recorded from the outlet of the cell using a mass spectrometer (MS). The experiments were performed at 100, 200, and 300 °C to study the effect of temperature on the activity/evolution of oxygen species. Argon was then replaced again by oxygen and the cycle (C_2_H_4_ + O_2_ → C_2_H_4_ + Ar → C_2_H_4_ + O_2_) was repeated 10 times. The spectra from the third to the tenth cycle were normalized by the total number of cycles to improve the signal-to-noise ratio. The time-resolved spectra, even after normalization by 8 cycles, remained excessively noisy, as depicted in Figure S16a. In response to this challenge, we developed a new data processing technique to distinguish between active and spectator species and extract the evolution of the active species from the original noisy data.

The phase sensitive detection (PSD) method, commonly employed in the analysis of time-resolved XRD, XAS, infrared, and XPS transient data, extracts sinusoidal signals from time-resolved data to analyze the kinetic information.^48−50^ Other studies tried to learn the responses at higher frequencies by designing modulation excitation experiments with square-shaped stimulation.^51,52^ Through this PSD method, signals corresponding to specific frequencies from 1 to 3 (see eq S1) can be extracted without interference from background species. Moreover, audio noise reduction, a typical signal processing technique, utilizes Fourier transform to analyze audio signals spectrally, identifying frequency components of noise for suppression and elimination. Inspired by these methods, a frequency-selective data analysis method (FSDA) has been developed to extract signals within a specific frequency range from time-resolved data, thereby filtering out influences from spectator species and noise.

The transient experiment generates time-domain curves, representing the intensity function at each collected O 1s binding energy over time. Each time-domain curve captures the signal variation at specific O 1s binding energies during the transient experiments. Utilizing the Fourier transform, these time-domain curves can be decomposed into the summation of sinusoidal signals with different frequencies, as shown in eq S1. The discrete Fourier transform breaks down the discrete digital signal from time-domain data into sinusoidal waveforms with varying frequencies. To achieve this decomposition, the fast Fourier transform function (FFT) is employed. When the frequency (*K*) is 0, the corresponding sinusoidal wave denotes the unchanging part of the signal, representing the spectator components that do not evolve during the process. A frequency of one corresponds to the part analyzed by the traditional PSD technique. Additional sinusoidal curves with frequencies 2, 3, and up to a half of the spectral number can be further extracted by the FFT function. The original time-domain curve can be reconstructed by summing the signals at all frequencies. In this process of FSDA method analysis, a summation of the sinusoidal waves extracted with the FFT function from the selective frequency range from 1 to a specific value is performed. Consequently, the sinusoidal curve with *K* = 0, representing the spectator species that are inactive during the transient process, is removed from the original signal. Additionally, signals with high frequencies, considered as noise in the spectra, are filtered out. The resulting extracted sinusoidal signals, spanning frequencies from 1 to an appropriate value, are then used to simulate the actual evolution of the active sites corresponding to a square wave excitation. Figure S16b–d displays the FSDA method processed time-resolved O 1s XPS data acquired at 300 °C. The purple curves indicate conditions where the silver foil sample is exposed to ethylene and oxygen, while the green curves represent scenarios where oxygen is replaced with argon at the same partial pressure. Figure S16b shows the curves (superimposed) extracted from the original data with a frequency of zero, representing the constant part of the signal within the investigated BE range. As illustrated in Figure S16c, when the extracted frequency is one, the evolution of the signal at each binding energy changes gradually in response to a sinusoidally shaped perturbation. A summation of the extracted time-domain curve from *K* of 1 to 4 is obtained using the FFT function, as shown in Figure S16d. If the frequency increases, the FSDA method processes the data with the real evolution but this becomes noisier. An optimal frequency has to be chosen within a range from 1 to a specific value, in order to allow the processed time-domain data to represent the real evolution of the active species while avoiding excessive noise that may hinder peak/s identification.

### In Situ Evolution of Oxygen Species under EPO Reaction Conditions

**Figure 5:**
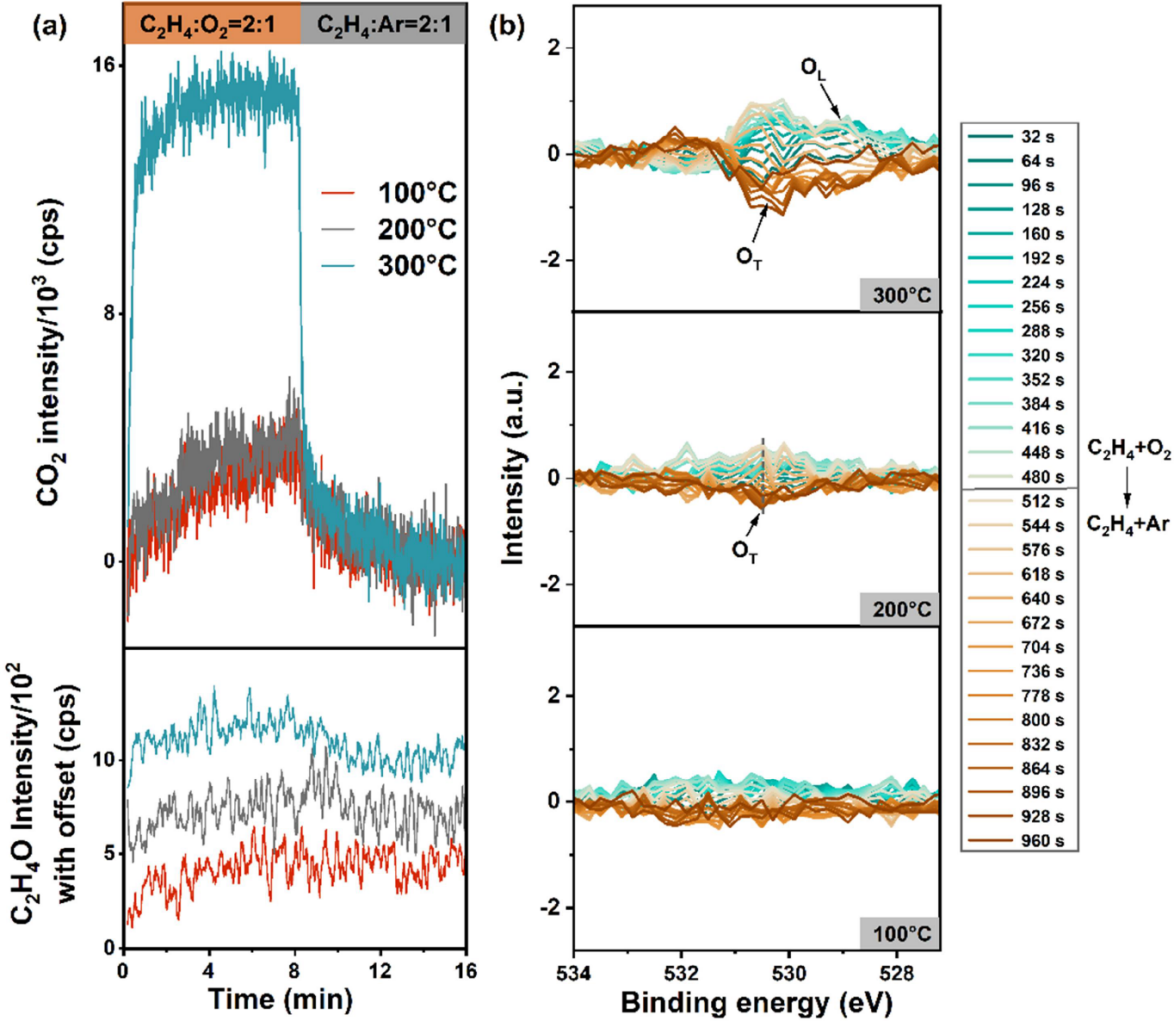
Evolution of products and active oxygen species in ethylene epoxidation transient experiments. (a) MS intensity of ethylene oxide and CO_2_ during the O_2_ cutoff transient experiments. (b) FSDA method processed in situ XPS results from the O_2_ cutoff transient experiments.

In recent years, significant efforts have been devoted toward understanding the role of different active oxygen species in ethylene oxidation. The emphasis was predominantly centered on identifying oxygen species selectively oxidizing ethylene to EO, as outlined in Table S7. The debate primarily concerns whether atomic oxygen or molecular oxygen serves as the selective species in the EPO reaction. Early studies supported atomic oxygen species as selective contributors to ethylene epoxidation.^53^ More recent research, employing Raman spectroscopy, supports the idea of molecular (dioxygen) species as the selective one toward EO formation.^2,54^ However, previous research mostly correlated the EO signal with the signal of oxygen species detected under steady state, without investigating the possibility to disentangle the reactivity of atomic oxygen from that of molecular oxygen. Through a combination of transient time-resolved experiments and the innovative FSDA technique, the results regarding reaction activity are presented in Figure 5. Figure 5a displays the MS signals of carbon dioxide and ethylene oxide collected over ten O_2_/Ar exchange cycles and normalized from the third to tenth cycle. It is important to highlight that the *m*/*z* = 43 was used for EO, to avoid overlaps with signals corresponding to other gases in the cell. However, *m*/*z* = 43 represents a minor fragment of EO in the cracking pattern.^55^ Simultaneously, the *m*/*z* = 44 was collected for carbon dioxide, which corresponds to the main fragment. At temperatures of 100 and 200 °C, minimal carbon dioxide signals are observed, with negligible EO intensity. Upon reaching 300 °C, the EO signal increases in the presence of oxygen, accompanied by a substantial increase in the intensity of carbon dioxide. Time-resolved O 1s spectra reported in Figure 5b were processed using the FSDA method with frequencies ranging from 1 to 4. The evolution of surface oxygen species, ranging from 529.0 to 533.0 eV, is minimal at 100 and 200 °C, with a slight evolution of the peak around 530.5 eV starting at 200 °C. At 300 °C, the fluctuation of surface oxygen species sharply increases, consistent with the MS signal. The most relevant evolution is detected at 530.5 eV and attributed to O_T_ species. We employed a second strategy to display changes of the O 1s during transient experiments. Upon averaging each single O 1s photoemission spectrum from consecutive cycles performed at the same temperature, we plotted and investigated the obtained 200 spectra per gas switch (C_2_H_4_ + O_2_ → C_2_H_4_ + Ar). Such an approach, which is based on previous literature,^56,57^ allows to improve the signal-to-noise ratio of each spectrum by event-averaging. Subsequently, we developed a batch processing procedure, which selectively deconvolutes each spectrum. The fitting parameters are listed in Table S8, and the fitting processes are shown in Videos 1, 2, and 3, corresponding to the experiments performed at 100, 200, and 300 °C, respectively. In each video, panel (a) displays the series of event-averaged O 1s photoemission spectra. The three-dimensional plot that is generated clearly displays the oxygen cutoff (oxygen replaced by argon in the reaction mixture) because the peaks corresponding to gas phase oxygen (536–540 eV BE) disappear from the spectrum. Panel (b) shows the fitting of a single event-averaged O 1s spectrum to be used as a guide for panel (c), which plots the trends of O_L_, O_T,_ and O_S_ with time. The three-dimensional panel (d) reports sum spectra (resulting from each O 1s deconvolution) with time. A clear decrease with time of O_T_ and O_L_ is observed upon oxygen cutoff. The trends of peak areas from O_L_, O_T,_ and O_S_ are also shown in Figure S17. Overall, the results agree well with those processed through the FSDA method. Therefore, oxygen atoms located at the topmost silver surface, derived from adsorbed dioxygen, are identified as the most active species under in situ EPO reaction conditions compared to other oxygen species. A small contribution centered at 529.1 eV, attributed to O_L_, suggests that this species also participates in the reaction, but with much lower activity than O_T_ species. Notably, even at 300 °C, a significant amount of carbon dioxide is produced alongside EO, indicating that O_T_ contributes to the formation of both products. This is consistent with previous studies making use of isotopically labeled oxygen.^3,58^ It is important to mention that EO could decompose to carbon dioxide on the surface of silver at 300 °C. Because the foil employed in this study is a poorly selective catalyst for EPO reaction and pressures in the mbar range also do not favor EO formation, the selectivity toward EO is much lower than that typically reported at 1 bar (around 50% on a Ag/Al_2_O_3_ catalyst).^1^ As an example, Rocha et al. carried out EPO reaction under similar pressure and temperature conditions as those of our study (0.3 mbar and 230 °C) but used silver powder as a model catalyst.^16^ The selectivity toward EO was always around/below 10%. Based on these assumptions, we can conclude that the majority of carbon dioxide detected in our work generates from ethylene combustion instead of ethylene oxide combustion. Consequently, dioxygen species emerge as the most active, challenging the notion of a selective species exclusively producing EO. The current results emphasize the paramount role of molecular oxygen adsorbed on silver surface oxide in the oxidation of ethylene. The coexistence of different types of adsorbed molecular oxygen species on the surface of silver suggests that their properties could be the key parameter to consider when discussing the selectivity toward EO.

## Conclusions

This study reveals the structure and dynamic behavior of active oxygen species on silver-based catalysts during the oxidation of ethylene. A combination of in situ/operando AP-XPS and DFT calculations leads to the identification of three distinct groups of oxygen species formed on the topmost layers of silver: lattice oxygen from surface oxide reconstructions, which has a nucleophilic behavior and top and subsurface oxygen derived from adsorbed dioxygen, which display an electrophilic behavior. Such species correlate well with silver oxide spectroscopic features detected in the Ag 3d signal, proposing a new method to indirectly follow the activation of oxygen on silver in actual (silver nanoparticles supported on an oxide) catalysts. A newly developed FSDA method is employed to process time-resolved data, enabling the discrimination and the detection of active species evolution by filtering out background signals and noise. The versatility of FSDA suggests its potential application in various time-resolved characterization methods in future studies. Notably, the top oxygen species from adsorbed dioxygen emerges as the most active, significantly influencing the catalytic activity. Furthermore, dioxygen is also proven not to be exclusively selective, contributing to the formation of both EO and carbon dioxide. These results suggest that dioxygen species adsorbed on the surface of silver have a paramount role in the oxidation of ethylene. Their different structures and properties will deserve more attention in future research aimed at understanding selectivity and activity patterns on doped catalysts that have been modified to maximize EO yield.

## Supporting Information Available

In situ characterization results (steady- and transient-state) and DFT calculations, schematic illustration of the Ag 3d spectra, O 1s spectra, a depth-profile analysis of O species, DFT models of oxygen species on Ag surface, detailed fitting parameters, and literature summary (PDF)
Video 1; fitting of event-averaged O 1s photoemission spectra (*t* = 100 °C) acquired in the transient experiment and fitting results (MP4)
Video 1; fitting of event-averaged O 1s photoemission spectra (*t* = 200 °C) acquired in the transient experiment and fitting results (MP4)
Video 1; fitting of event-averaged O 1s photoemission spectra (*t* = 300 °C) acquired in the transient experiment and fitting results (MP4)
