# Supplementary material for: Structure and Reactivity of Active Oxygen Species on Silver Surfaces for Ethylene Epoxidation
Source: ACS Catal. 2024 Jun 21;14(13):10234–44. doi: 10.1021/acscatal.4c01566 (PMC11232021; doi:10.1021/acscatal.4c01566)
Supplement: Supplementary file 1 — cs4c01566_si_001.pdf [file cs4c01566_si_001.pdf]

Supporting Information

Structure and Reactivity of Active Oxygen Species on Silver Surfaces for Ethylene Epoxidation

Man Guo[1,2,#], Nanchen Dongfang[3,#], Marcella Iannuzzi[3], Jeroen Anton van Bokhoven[1,2], Luca Artiglia[1,]*

[1] Laboratory for Catalysis and Sustainable Chemistry, Paul Scherrer Institute, 5232 Villigen, Switzerland.

[2] Department of Chemistry and Applied Biosciences, Institute for Chemical and Bioengineering, ETH Zurich, 8093 Zurich, Switzerland.

[3] Department of Chemistry, University of Zurich, 8057 Zurich, Switzerland.

[#] Contributed equally to the writing of the manuscript.

* Luca Artiglia luca.artiglia@psi.ch

Table S1. Literature review of the oxygen species and their B.E. on Ag surface by XPS.

	Sample	Gas conditions	Temperature	BE (eV)	Oxygen species	Reference
1	Ag powder	5%C_2H_4+5%O_2 in 1 mbar (in-situ)	25°C -300°C	~532.0	Surface Ag_x-O_2 dioxygen species	ACS Catal. 2022, 12, 4375−4381
				~530.0	Atomic O_{bulk} species	
2	Ag foil	Vapor phase pretreatment for 2 h (ex-situ)	800°C	531.1	Molecular forms of oxygen	SEMICONDUCTORS Vol. 53 No. 15 2019
				530.3	Atomic oxygen in Ag_2O	
		Oxygen ions pretreatment for 2 h		532.2	Molecular oxygen	
				530.5	Atomic oxygen on the surface	
				529.8	Ag_2O oxide	
3	Ag (111)	NO_2 pretreatment (ex-situ)		~530.2	O_{elec}; adsorbed SO_4; the only species shown to produce EO	ACS Catal. 2018, 8, 3844−3852
				528.4	O_{nuc}	
4	Ag (110)	O_2 in 10^{-6} to 10^{-5} mbar	150°C	≤ 528	Unreconstructed adsorbed atomic oxygen (O_{ads}); present at very low O-converages that slightly higher than UHV at low temperature.	Chem. Sci.,2018, 9, 990
				528.2	Reconstructed oxygen from p(N*1)	
		O_2 in 10^{-7} to 10^{-6} mbar	-150°C - 180°C	529.7	Molecularly adsorbed oxygen	
				~528	Unconstructed atomic oxygen	
5	Ag (111)	O_2 (1-100 pa) pretreatment for 10 min (ex-situ)	27°C -147°C	530.0	Oxygen adsorbed at the surface, not from carbonates; occupy the topmost layer	J. Chem. Phys., Vol. 111, No. 5, 1 August 1999
				528.2	Adsorbed atomic oxygen-O_α; oxygen	

					between the top and second silver layers	
6	p(4*4) reconstruction on Ag(111)	Pretreated by NO_2, and then exposed to C_2H_4 at 0.1 mbar for 10 min (ex-situ)	25°C	531.65	Readsorbed NO/NO_2	J. Phys. Chem. C 2016, 120, 28630–28638
				530.5	formate	
				528	Oxygen on the reconstructed surface	
7	Ag (111)	O_2 at 0.3 torr (in-situ)	RT to 140 °C	531.5	OH-groups	Surface Science 652 (2016) 51–57
				530.2	Disordered atomic oxygen	
				528.9	Oxygen in Ag oxide (at step edges)	
				528.1	Oxygen in the Ag(111)-p(4*4)-surface reconstruction	
8	Ag (111)	O_2 in $4*10^{-4}$ mbar (in-situ)	150°C	527.95	Unreconstructed atomic oxygen which is likely not present in EPO conditions.	Phys. Chem. Chem. Phys., 2015, 17, 9288--9312
				528.3	Oxygen atoms from p(4*4) phase	
				529.1	Oxygen from Ag_2O oxide	
9	Ag nanopowder	C_2H_4:O_2=1:2 (0.3 mbar, EtCl pulses, in-situ)	230°C	528.3-528.6; 529.1-529.3	Nucleophilic oxygen species	Journal of Catalysis 312 (2014) 12–16
				530.1-530.6; 530.9-531.1	Electrophilic oxygen species	
10	Ag (111)	Atomic oxygen ($5*10^{-7}$ – $1*10^{-6}$, ex-situ)	227°C	528.2	Oxygen from p(4*4) and c(4*8)	J. Phys. Chem. C 2014, 118, 15324–15331
				528.7	Ag_2O > 1 layer	
				530.0	OH	

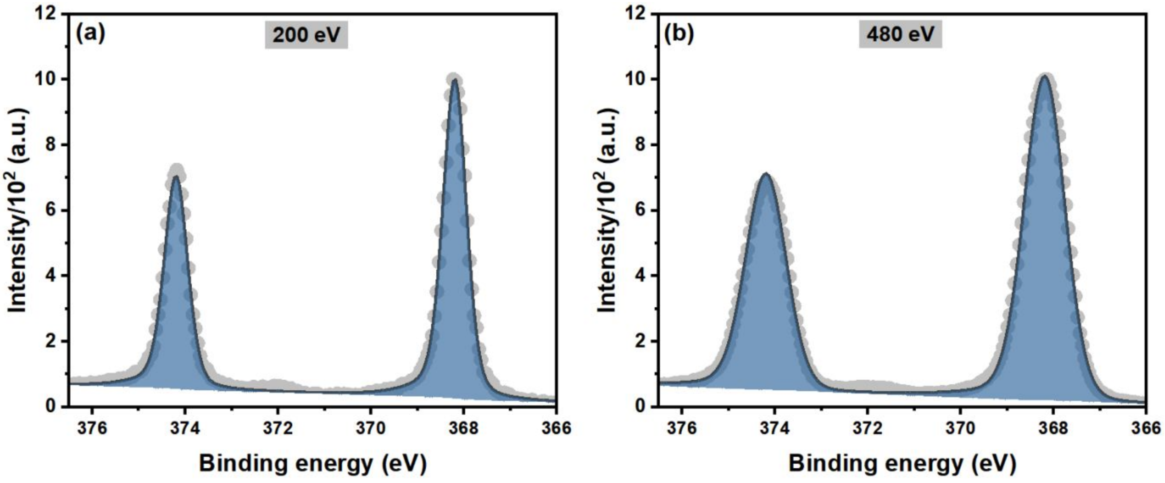

Figure S1. Ag 3d reference spectra collected from 400°C in high vacuum at 200 eV (**a**) and 480 eV (**b**).

Table S2. Fitting parameters of Ag 3d peaks from the reference spectra.

	B.E. (eV)	FWHM (eV)	%L-G	Asymmetry parameters[a]
200 eV	368.2	0.6	28	0.03, 1.2
480 eV	368.2	1.0	9	0.03, 1.2

Table S3. Fitting parameters of Ag 3d peaks (K.E.=480 eV).

	B.E. (eV)	FWHM (eV)	%L-G	Asymmetry parameters
Ag_0	368.2	1.0	9%	0.03/120
Ag_α	367.8	1.0	9%	0
Ag_β	367.4	1.0	9%	0

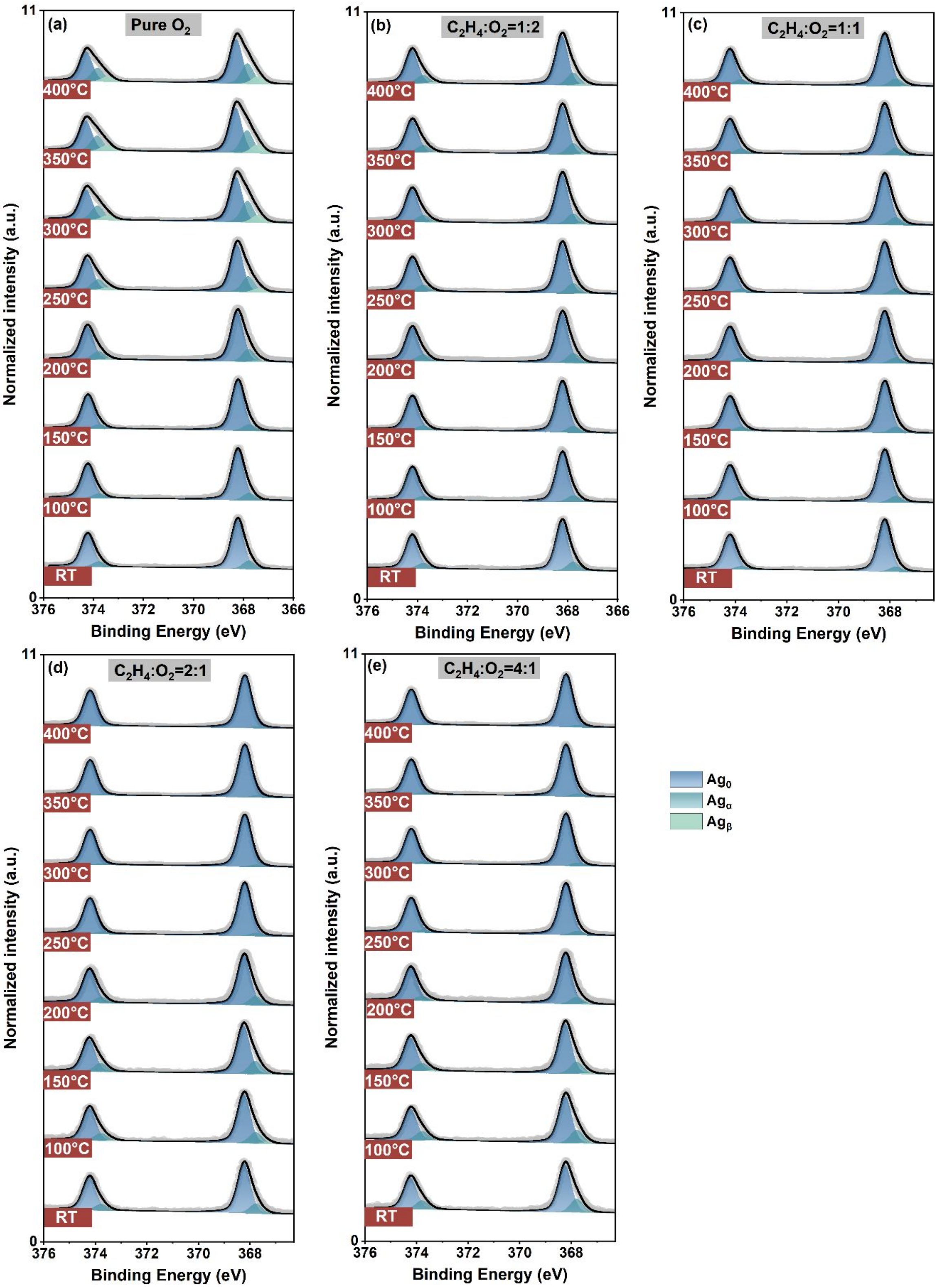

Figure S2. a-e. Ag 3d fitting spectra collected at a kinetic energy of 200 eV while exposing the sample to different reaction environments and temperatures.

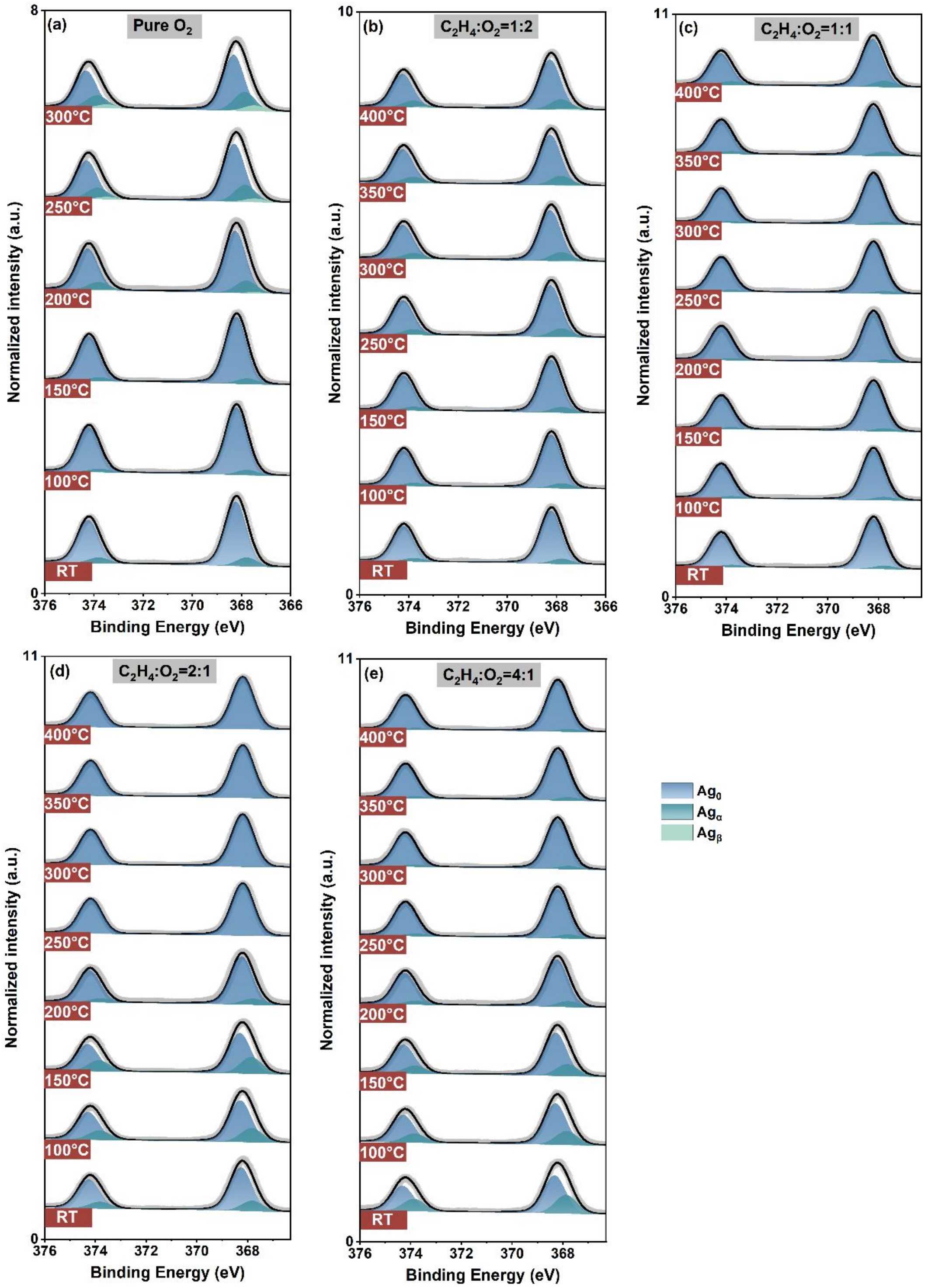

Figure S3. a-e. Ag 3d fitting spectra collected at a kinetic energy of 480 eV while exposing the sample to different reaction environments and temperatures.

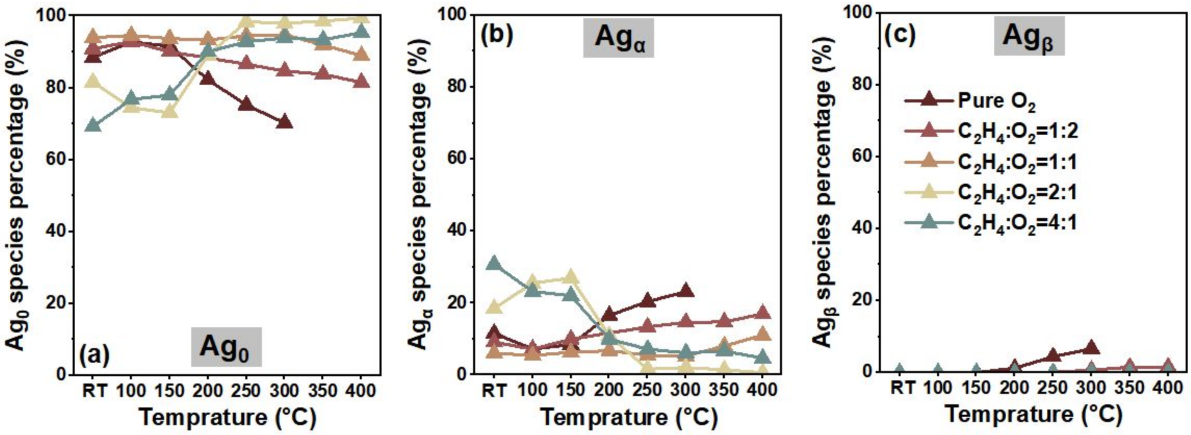

Figure S4. Fraction of Ag_0 (**a**), Ag_α (**b**) and Ag_β (**c**) in different reaction environments as a function of temperature (KE=480 eV).

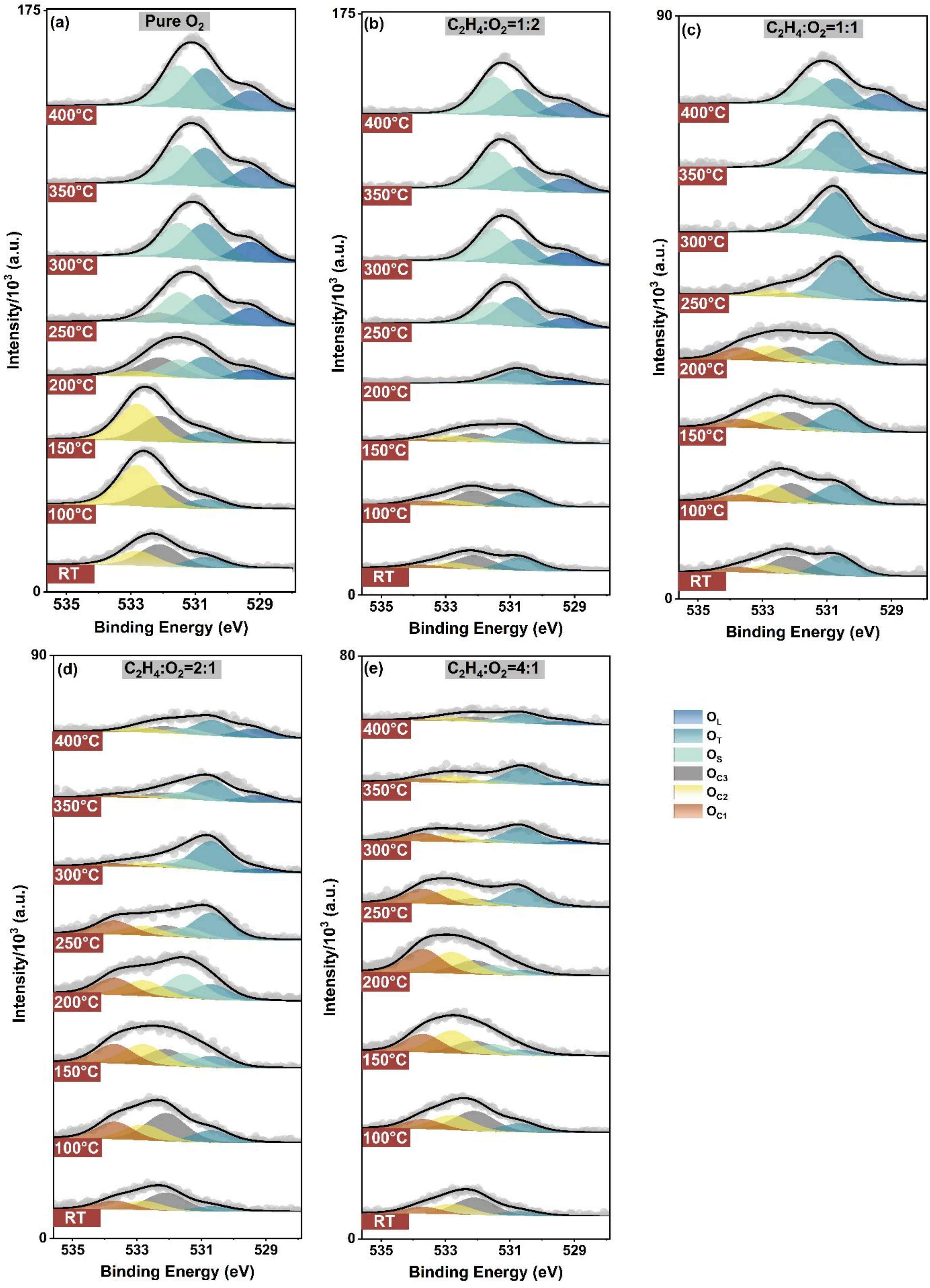

Figure S5. a-e, O 1s fitting spectra collected at different environment (KE=200eV).

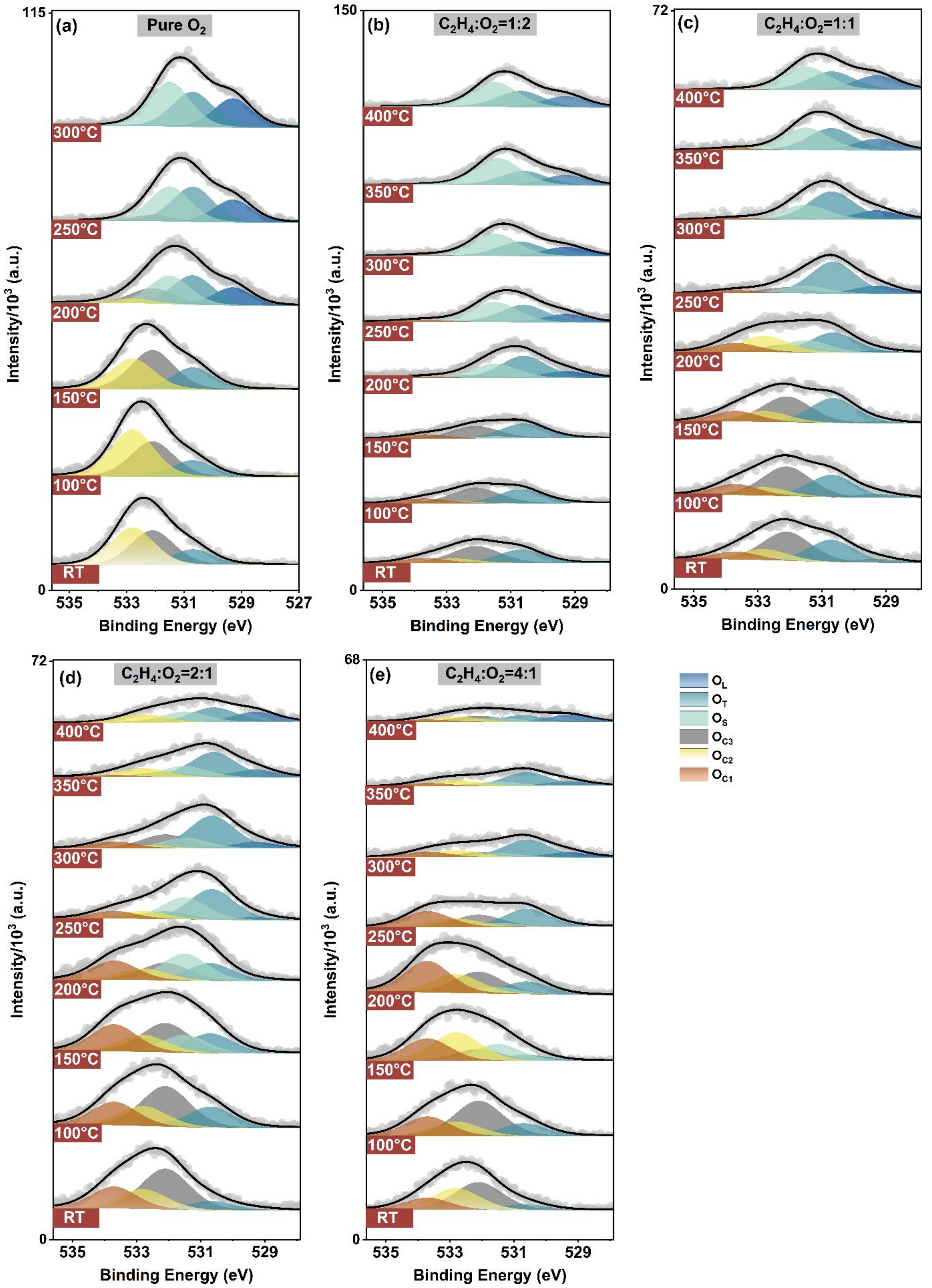

Figure S6. a-e, O 1s fitting spectra collected at different environment (KE=480eV).

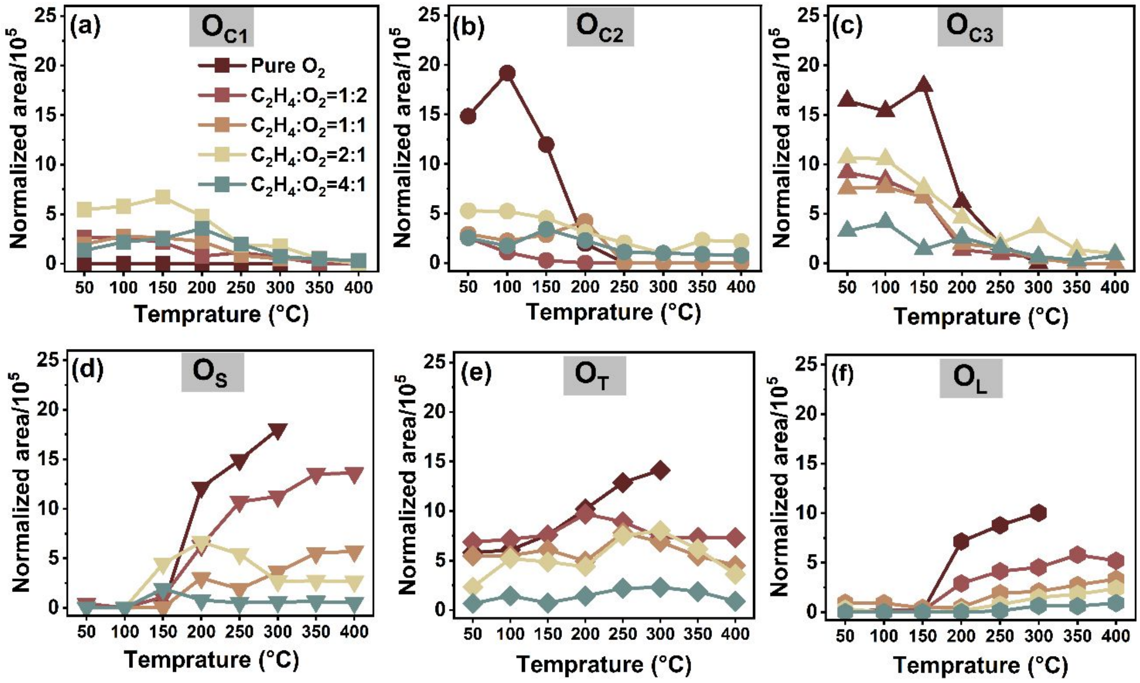

Figure S7. a-f, Fraction of O_{C1} (**a**), O_{C2} (**b**), O_{C3} (**c**), O_S (**d**), O_T (**e**) and O_L (**f**) in different reaction environment as a function of temperature (K.E.=480 eV).

Table S4. Fitting parameters of O 1s peaks (KE=480 eV).

	B.E. (eV)	FWHM (eV)	%L-G
O_{C1}	533.7	1.7	25%
O_{C2}	532.8	1.8	25%
O_{C3}	532.1	1.8	25%
O_S	531.5	1.7	25%
O_T	530.7	1.7	25%
O_L	529.3	1.6	25%

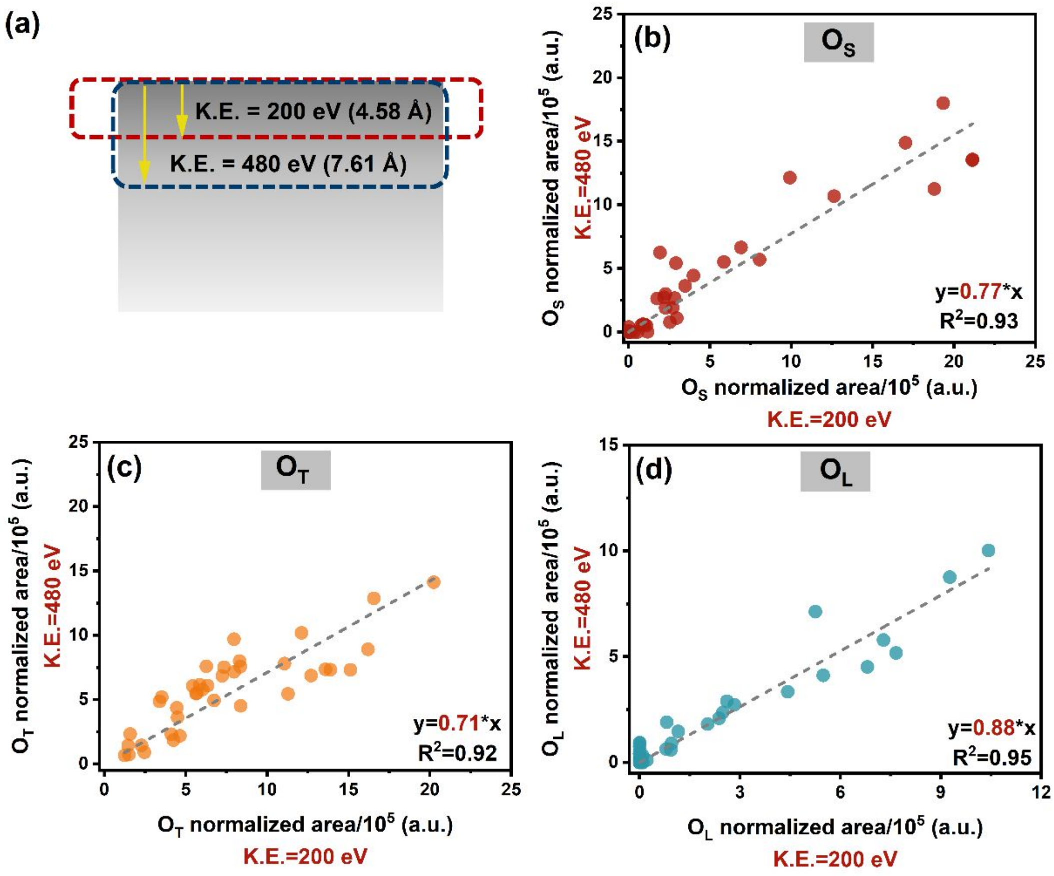

Figure S8. a, Schematic illustration of the detection depth when the kinetic energy is 200 eV and 480 eV, respectively. **b-d**, Correlation of the normalized peak areas of O_S (**b**), O_T (**c**) and O_L (**d**) between K.E. of 200 eV and K.E. of 480 eV.

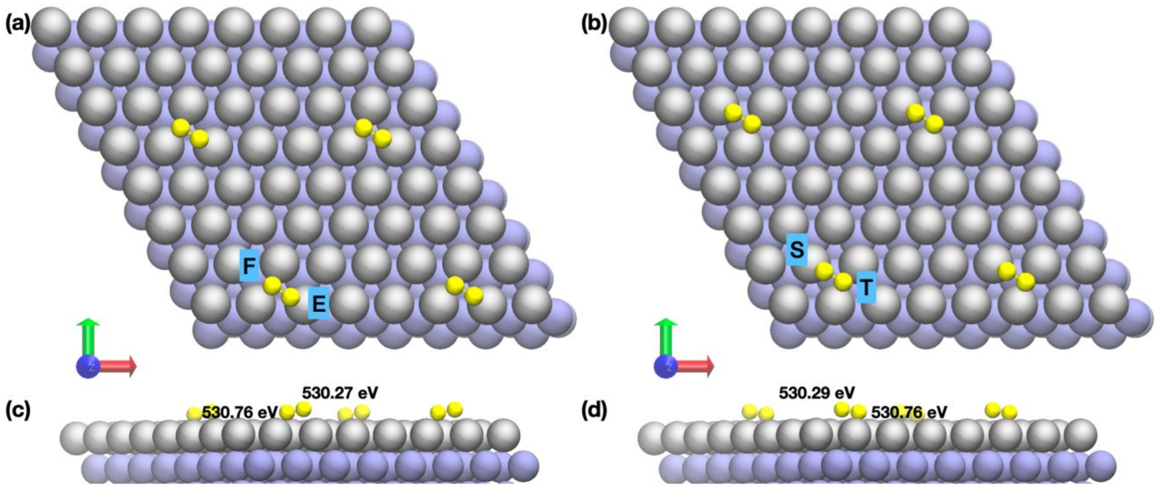

Figure S9. a, c Calculated BE of molecular oxygen at FCC sites and **b, d** HCP sites of Ag (111) surface

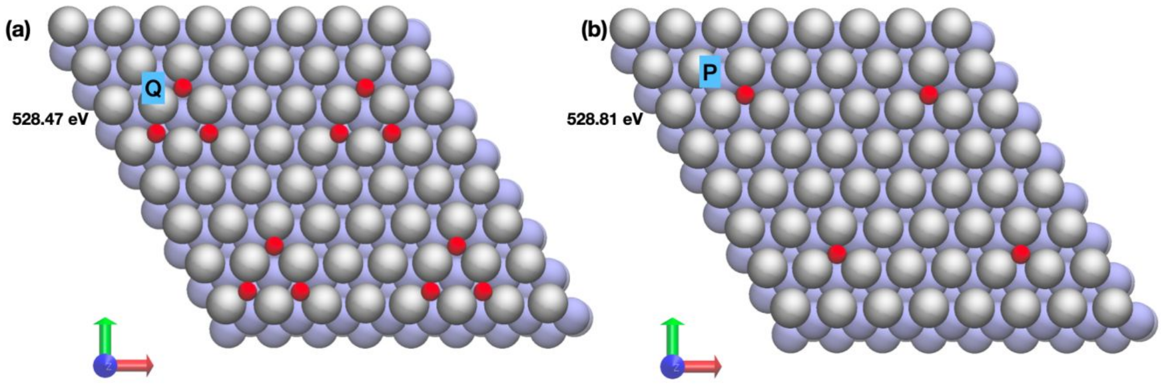

Figure S10. Calculated BE of atomic oxygen at the FCC sites on unreconstructed Ag (111) surface

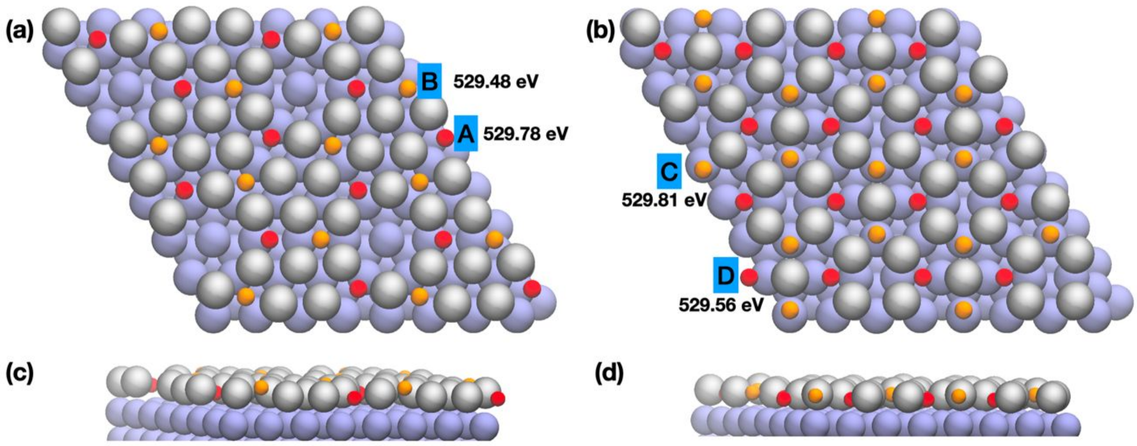

Figure S11. a, c Calculated BE of surface lattice oxygen on p (4x4) and **b, d** c (4x8) reconstructed surface.

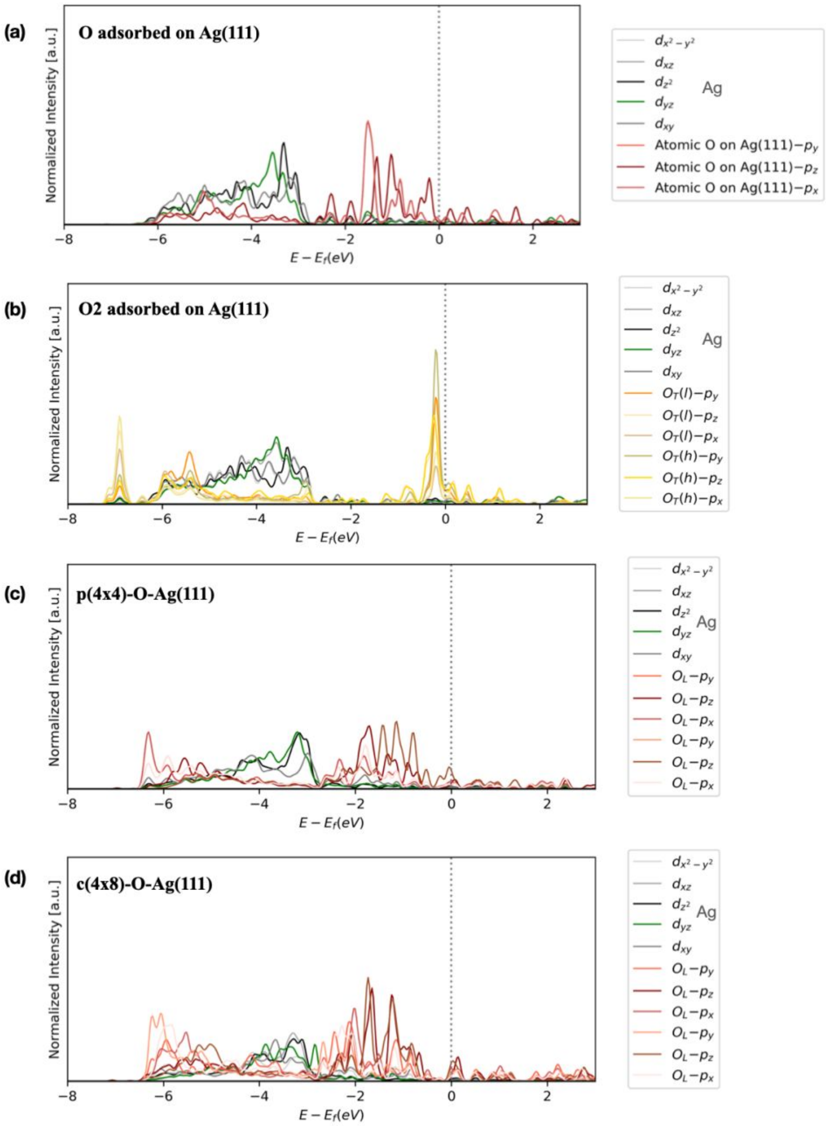

Figure S12. Projected DOS for simulated models: (a) atomic oxygen adsorbed on clean Ag(111) surface, (b) molecular oxygen adsorbed on clean Ag(111) surface, (c) p(4x4) oxidized reconstruction silver surface, (d) c(4x8) oxidized reconstruction silver surface.

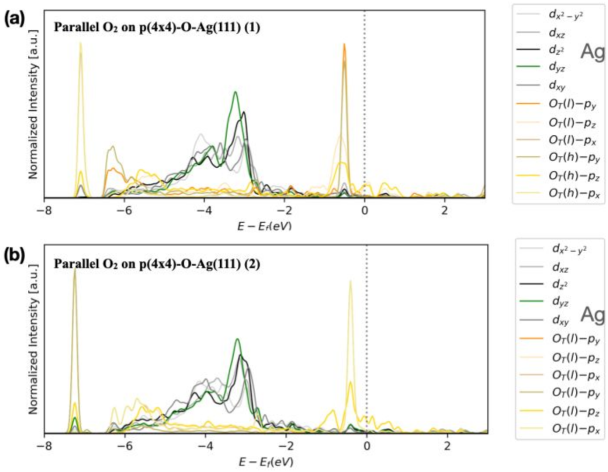

Figure S13. Projected DOS for simulated models: (a) parallel O_2 on p(4x4) oxidized reconstruction Ag(111); the structure shown in Figure 4(b); (b) parallel O_2 on p(4x4) oxidized reconstruction Ag(111); the structure shown in Figure 4(c).

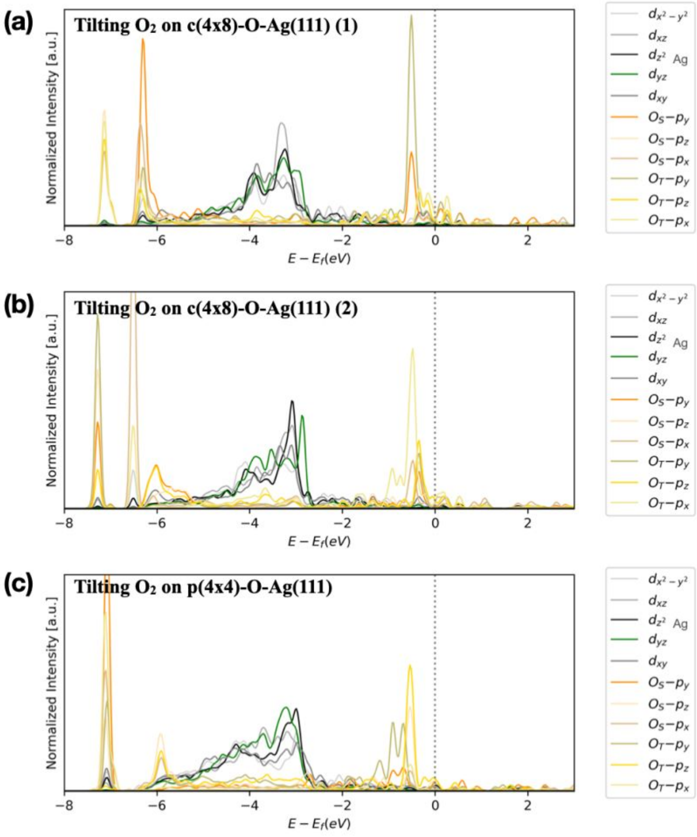

Figure S14. Projected DOS for simulated models:(a) tilting O_2 on c(4x8) reconstruction silver surface(1), its structure shown in Figure 4(e); (b) tilting O_2 on c(4x8) reconstruction silver surface(2), its structure shown in Figure 4(f); (c) tilting O_2 on p(4x4) reconstruction silver surface, its structure shown in Figure 4(d).

Table S5. Comparison between the calculated and experimentally observed BE of different oxygen species.

	BE (eV) from XPS experiment	BE (eV) from DFT[b]	ID (O species)	Slope[a]
O_S	531.5	531.74-531.83	K, M, O	0.69
O_T	530.7	530.27-531.31	E, F, G, H, J, L, N	0.60
O_L	529.3	529.48-529.81	A, B, C, D	0.85

[a]The slopes are from the linear correlation between the oxygen species of two K.E. as shown in Fig. 3b–d.

[b]All calculations of BE, was achieved by applying the Gaussian augmented plane wave method (GAPW) in CP2K. For the specific case of the implementation of the method in CP2K, there are some tutorials on the CP2K web-site [https://www.cp2k.org/exercises:2019_conexs_newcastle:index].

Table S6. Summary of the structural information for calculated oxygen species at possible steady-state conditions on silver-based surfaces.

ID	Species	E_{ads} (eV) / per O	B.E. (eV)	Mulliken Charge population	Atomic Structures
A/B	p(4x4)-O-Ag(111)	-	529.78/528.48	-0.52/-0.54	Figure S11 a,c
C/D	c(4x8)-O-Ag(111)	-	528.81/528.56	-0.56/-0.49	Figure S11 b,d
E/F	O_2/fcc site	-0.35	530.27/530.76	-0.19/-0.21	Figure 4 a and S10 a,c
G/H	p(4x4)-O-Ag(111)-O_2-parallel (1)	-0.52	531.31/530.98	-0.28/-0.27	Figure 4 b
I	p(4x4)-O-Ag(111)-O_2-parallel (2)	-0.40	530.97	-0.26	Figure 4 c
J/K	p(4x4)-O-Ag(111)-O_2-tilting	-0.55	530.99/531.83	-0.29/-0.32	Figure 4 d
L/M	c(4x8)-O-Ag(111)-O_2-tilting(1)	-0.34	530.36/531.81	-0.23/-0.24	Figure 4 e
N/O	c(4x8)-O-Ag(111)-O_2-tilting(2)	-0.58	530.59/531.74	-0.24/-0.28	Figure 4 f
P	O/fcc site	-0.50	528.81	-0.49	Figure S9 b
Q	O/3_fcc site	-0.39	528.47	-0.45	Figure S9 a
R	O/hcp site	-0.41	527.74	-0.49	Figure S16 a
S/T	O_2/hcp site	-0.33	530.29/530.76	-0.19/-0.21	Figure S11 b,d
U/V	O-AgO_2-Ag(111)	-	529.13/529.19	-0.53/-0.53	Figure S16 b
W/X	O-Ag_4O_4-Ag(111)	-	528.30/529.39	-0.51/-0.52	Figure S16 c

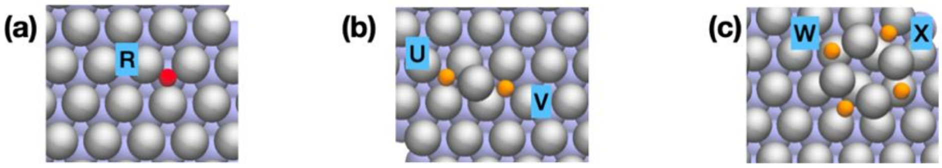

Figure S15. (a) The calculated model for atomic oxygen at HCP site on clean Ag(111) surface, (b) AgO_2 forming on clean Ag(111) surface, (c) Ag_4O_4 forming on clean Ag(111) surface. In (b), the difference between O labeled by U and V is their locations, at HCP site and FCC site with respect to the Ag(111) surface, respectively. In (c), the difference between O labeled by W and X is their locations at top site and bridge site with respect to the Ag(111) surface.

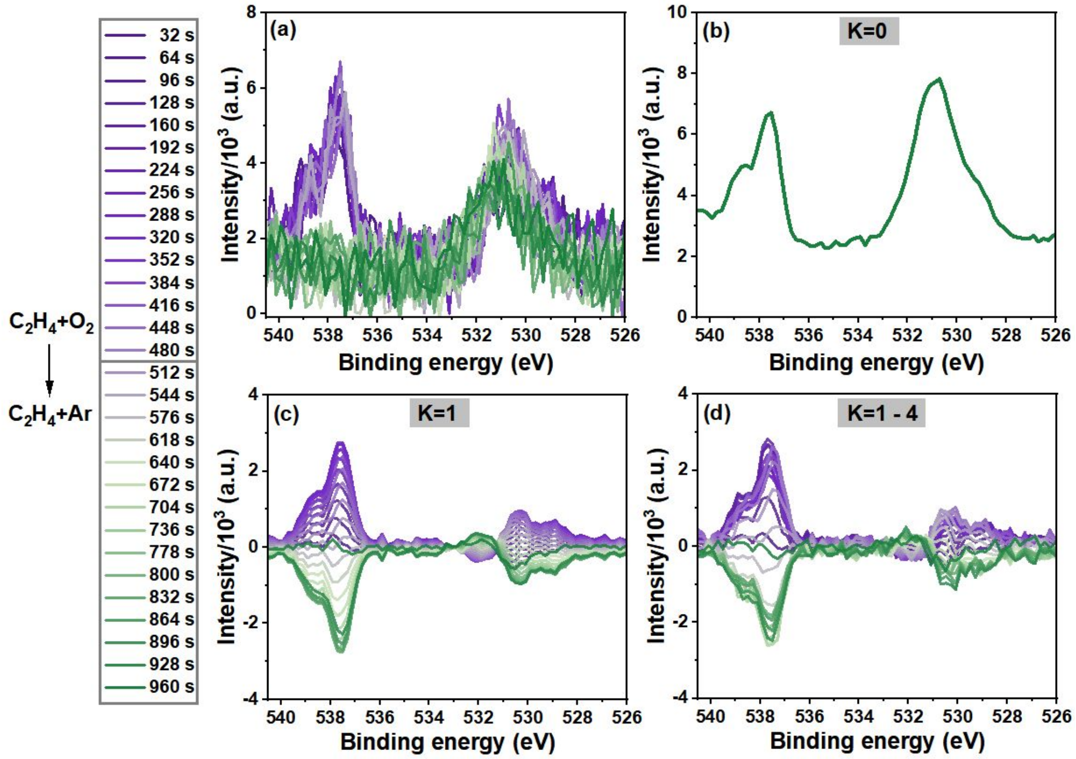

Figure S16. a, Original O 1s X-ray photoelectron spectra collected during the transient experiments at 300°C. **b-d**, FSDA processed results at different frequencies.

$$x_n = \sum_{k_0}^{k_n} X_k e^{i\frac{2\pi}{N}kn} \qquad n = 0, \ . \ . \ . \ , N-1 \qquad (S1)$$

n: Data point of the time resolved curve at a specific B.E..

N: The total number of data points of the time-resolved curve at a specific B.E..

x_n: Processed intensity using the FSDA method when the data point is n.

k: frequency.

k_0: The initial frequency for FSDA processing.

k_n: The final frequency for FSDA processing.

X_k: The amplitude calculated using Fast Fourier Transform (FFT) when the frequency is k.

Table S7. Literature review of the oxygen species on silver surface at various conditions.

	Material	Method	Gas conditions	Temperature	Significant conclusions about oxygen species	Reference
1	Ag nanoparticles	In-situ Raman, AP-XPS, DFT	1 bar (5% C_2H_4+5%O_2)	50°C-300°C	Ag_4-O_2 molecular oxygen species selectively oxidize ethylene to EO.	ACS Catal. 2022, 12, 4375−4381
2	Curved Ag (111)	STM	Gas-phase atomic oxygen	<200°C	Formation of adsorbed atomic oxygen on p(4*4) and p(4*5√3) reconstructed surface.	J. Vac. Sci. Technol. A 2021, 39, 053201
3	Ag/Al_2O_3	Ex-situ Raman, DFT	1 bar (10% O_2 pretreated)	400°C, 500°C	Hybrid oxygen atom reacts with one lattice oxygen atom on the surface or in the subsurface of Ag. This is the most selective species towards EPO.	Langmuir 2021, 37, 11603−11610
4	Ag nanoparticles	In-situ Raman	1 bar (C_2H_4+O_2 mixture)	150°C-400°C	Dioxygen complexes that are partially embedded in an oxide-like overlayer coexist with atomic oxygen on the silver surface during exposure to the reaction mixture.	J. Catal. 2022, 405, 445–461
5	Ag (111)	In-situ XPS, IR	10 mbar		O 1s peak between 530 eV and 531 eV is partly due to the surface carbonate and partly due to the electrophilic oxygen (no clear structure provided) that leads to EO formation.	Physchem 2021, 1, 259-271
6	Ag (111)	STM, LEED	High vacuum, gas-phase oxygen atoms	<500 K	A single oxygen species initially forms on the surface. At higher oxygen coverages, a new, three-dimensional oxide-like phase develops	J. Phys. Chem. C 2020, 124, 1382−1389
7	Ag (100)	DFT			Adsorbed ozone species (0.75 ML coverage) interpreted as a possible structure of electrophilic oxygen	Surface Science 2019, 679, 188–195
8	Ag foil	XPS	Water vapor	800°C	O 1s peak at 532.2 eV BE is assigned to molecular oxygen. The peak at 530.5 eV is assigned to atomic oxygen on the topmost layers. A BE of 529.8 eV is from Ag_2O oxide	Semiconductors 2019, 53, 1983–1985

9	Ag (111)	Ex-situ XPS	NO_2 pretreated		Electrophilic oxygen at 530.2 eV, the only species that has been shown to produce EO, is assigned to adsorbed SO_4.	ACS Catal. 2018, 8, 3844–3852
	Ag powder	In-situ XPS	0.3 mbar C_2H_4/O_2 mixture			
10	Ag (111)	DFT			Ag (111) is the most stable facet that maintains very low oxygen coverage. Ag (111) is the least active surface and only moderately selective in EPO reaction.	J. Chem. Sci. 2018, 130, 30

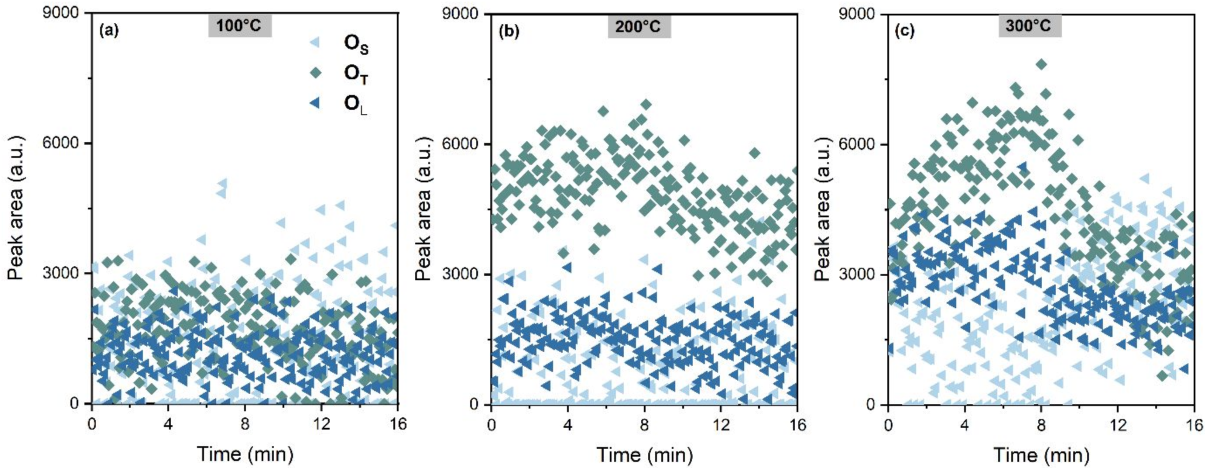

Figure S17. Fitting results of time resolved XP spectra at (a) 100°C, (b) 200°C and (c) 300°C.

Table S8. Fitting parameters of O 1s peaks (KE=270 eV) acquired during transient experiments.

	BE (eV)	FWHM (eV)	%L-G
O_S	531.5	1.6	25%
O_T	530.7	1.6	25%
O_L	529.3	1.5	25%
